# Supplementary material for: CRISPR/Cas9 targeting events cause complex deletions and insertions at 17 sites in the mouse genome
Source: Nat Commun. 2017 May 31;8:15464. doi: 10.1038/ncomms15464 (PMC5460021; doi:10.1038/ncomms15464)
Supplement: Supplementary Information — Supplementary Figures, Supplementary Tables, Supplementary Notes, Supplementary References. [file ncomms15464-s1.pdf]

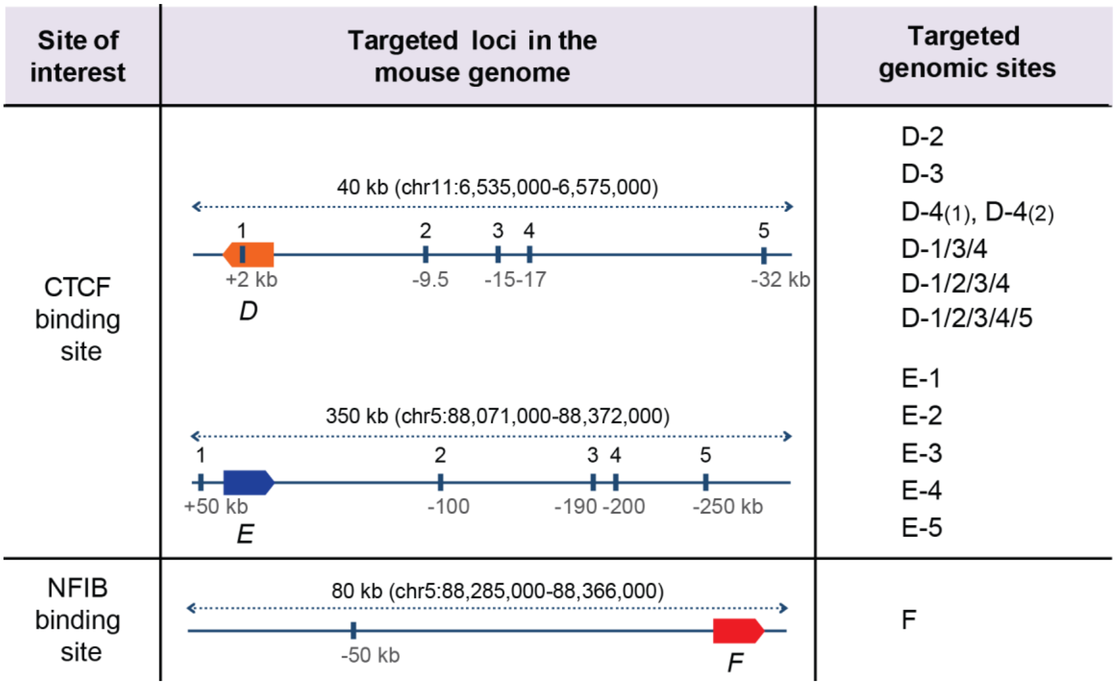

Supplementary Figure 1 Schematic diagram of CTCF and NFIB binding sites targeted by CRISPR/Cas9. Five CTCF binding sites in *Wap* locus (D), five CTCF sites in *Csn* locus (D) and one NFIB binding site in *Csn* locus (E) were targeted. The five sites in *Wap* locus were targeted separately or in combination as illustrated on the right panel. CTCF and NFIB binding sites in *Csn* locus were separately targeted. Arrowheads indicate the coding genes. The genomic coordinates and the distances of target sites to the respective gene are also shown.

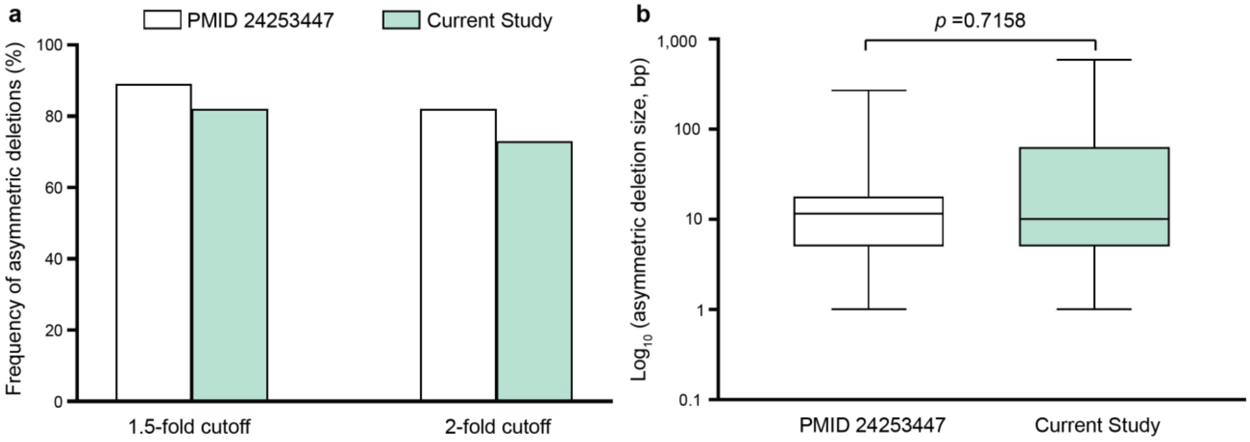

Supplementary Figure 2 Comparison of asymmetric deletions between a prior study (PMID 24253447) and the current study. (a) Frequency of asymmetric deletions observed in a prior study and the current study. Deletion sequences were obtained from the founder sequences of prior study (Supplementary Table 3 and 4)[1] and the current study. Subsequently, asymmetric deletions were examined with two cutoffs, 1) ≥ 1.5-fold difference between the upstream and downstream of Cas9 cutting site and 2) ≥ 2-fold difference between each side. The frequency of asymmetric deletions was prevalent in both studies (1.5-fold cutoff: 89% vs 82%, 2-fold cutoff: 82% vs 73%). In a prior study, deletion sequences of total 84 founders from two targeted genomic sites were analyzed. In the current study, deletion sequences of total 139 founders from nine targeted genomic sites were analyzed. Only deletions obtained from a single sgRNA injection were analyzed to avoid the effect of multiple variables. Deletions obtained from a single sgRNA injection in a prior study, Deletions targeting *Foxn1* and *Prkdc*; Deletions obtained from a single sgRNA injection in the current study, Deletions targeting TF binding site B, C-1, C-2/3, D-2, D-3, D-4(1), and F. (b) Deletion sizes of asymmetric deletions (1.5-fold cutoff) obtained from a prior study and the current study. Although the median deletion size of asymmetric deletions from the current study (10 bp) was similar to that from a prior study (11.5 bp), the top 50% of asymmetric deletion sizes was bigger in the current study (up to 600 bp). Median, middle bar inside the box; IQR, 50% of the data; whiskers, 1.5 times the IQR.

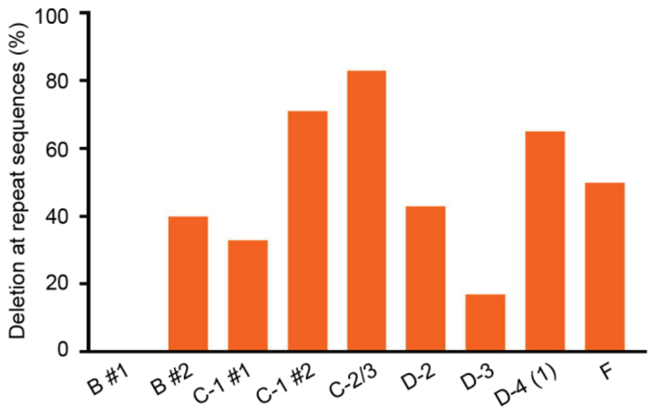

Supplementary Figure 3 Frequency of single sgRNA-imposed deletions found at repeat sequences. More than 80% of deletions were found at the repeat sequences with a single sgRNA targeting the genomic site C-2 in mice carrying a C-3 deletion (C-2/3). B #1, *n*=8; B #2, *n*=10; C-1 #1, *n*=6; C-1 #2, *n*=7; C-2/3, *n*=12, D-2, *n*=40; D-3, *n*=12; D-4(1), *n*=17; F, *n*=10.

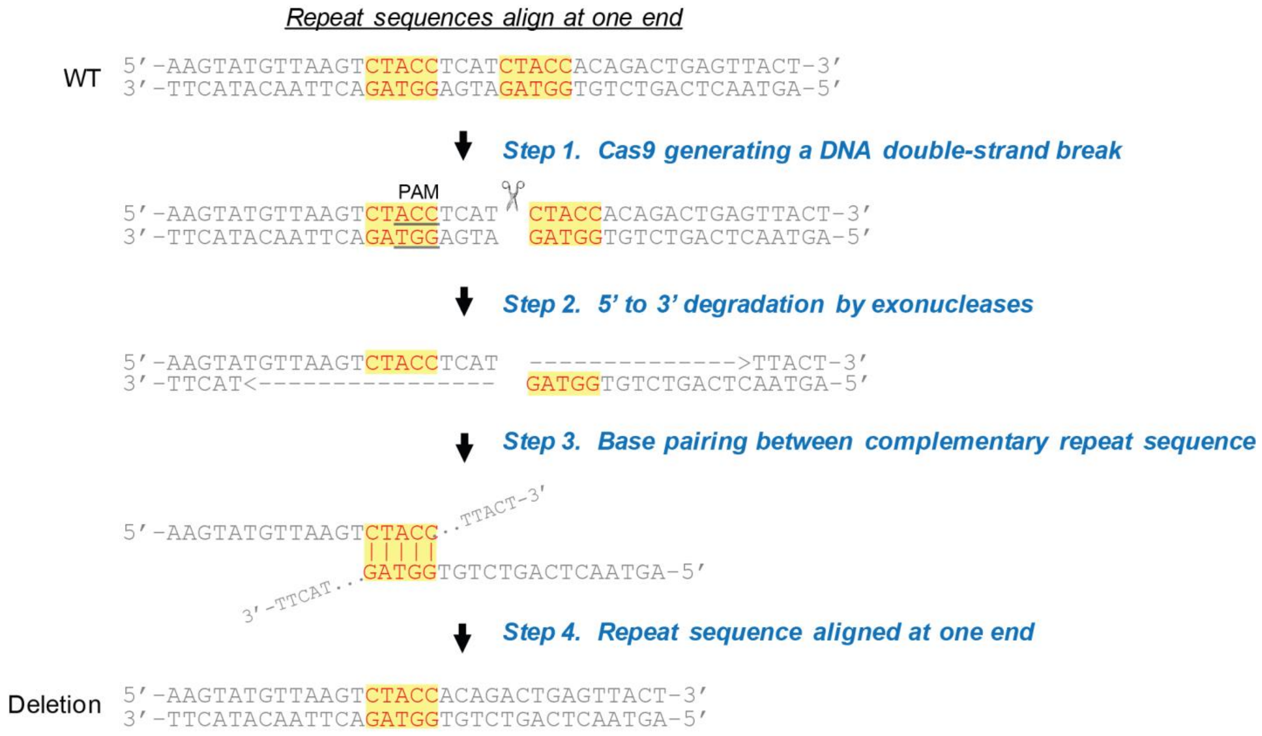

Supplementary Figure 4 Flow of proposed mechanism that can create repeat sequences aligned at one end. After Cas9 creates the blunt end cut 3 bp upstream of the PAM sequence, 5’ to 3’ exonucleases generate a deletion toward 3’ direction. Complementary repeat sequences generate the base pairing and DNA polymerase fills in the gap. Only a single unit of repeat sequence is retained.

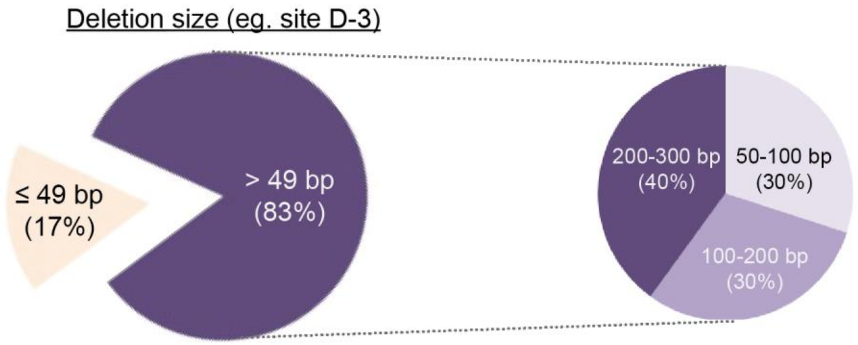

Supplementary Figure 5 Deletion sizes obtained from founders that were targeted at genomic site D-3 with a single sgRNA. More than 80% of deletions were larger than the average deletion size (49 bp). Among them, approximately 40% of deletions exceeded 200 bp.

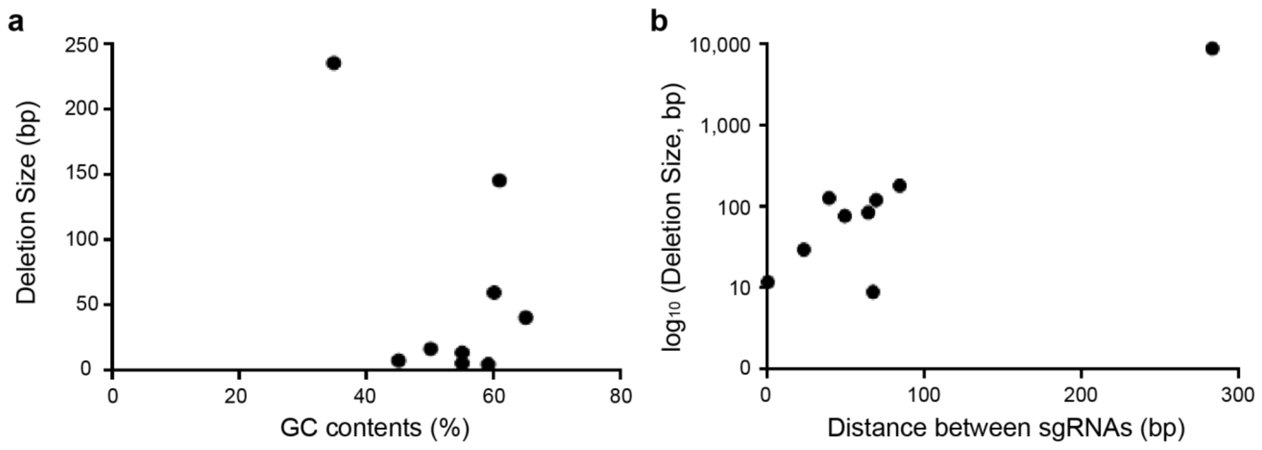

Supplementary Figure 6 Deletion sizes in relation to the GC content of sgRNAs and the distances between sgRNAs. (a) Scatterplot showing the deletion sizes dependent on the GC content of sgRNAs from injection of single sgRNAs. (b) A scatterplot depicting the deletion sizes dependent on the distances between each sgRNAs from co-injection of more than one sgRNAs targeting a single site.

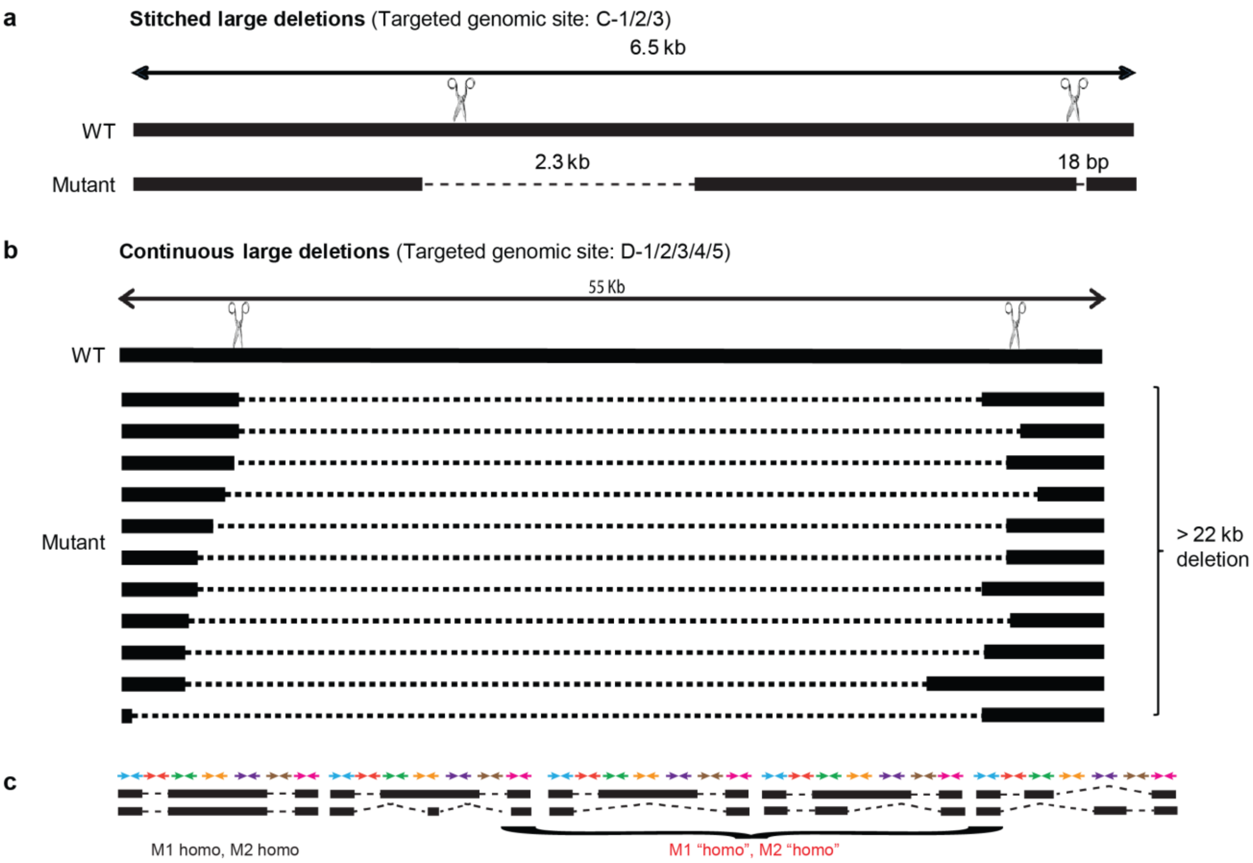

Supplementary Figure 7 Representative examples of large deletions obtained from the simultaneous injection of four sgRNAs corresponding to two distant sites and the strategy to identify compound large deletions. (a) Stitched large deletions in mice from site C-1/2/3. (b) Continuous large deletions in mice from site D-1/2/3/4/5. (c) Diagram depicting the genotyping method used to identify compound large deletions. Serial primers spanning the target region were used for the PCR genotyping.

Supplementary Table 1 List of studies on CRISPR/Cas9 genome editing in the mouse germline and studies with microhomology-based deletions

1. Deletion at repeat sequences

PMID	System (*in vivo* mouse or *in vitro* cell culture)	Deletion at repeat sequences: Repeat sequences aligned at 1) one end (or microhomology-mediated end joining) 2) both ends
Current study	in vivo mouse	1, 2
26887046	in vivo mouse	1 *no statistical analysis was done.
24972169, 24973878, 25201414, 25757625, 26787905, 27301063	in vitro cell culture	1
23709638, 27543296	Drosophila	1
26318124	Zebrafish	1
26199327, 25550322, 26573820	parasite	1

2. Large deletion by CRISPR-single sgRNA injection *in vivo* mouse

PMID	Large deletion (> 400 bp)
Current study	single (600 bp)
26887046	900 bp *no statistical analysis was done.

** 1 out of 18 refs (including PMID 24494965, 26501274, 23545779, 24253447, 24372541, 24675426, 25538209, 25819794, 26160899, 26442875, 26456390, 26713866, 26857612, 26936824, 26964564, 27139777, and 27246398) provided the information of large deletion generated by single sgRNA injection.

3. Large deletion by CRISPR-multiple sgRNA injection *in vivo* mouse

PMID	Large deletion (> 400 bp)
Current study	multiple (24 kb)
27119535, 23992847, 24984260, 26501274, 27151215, 27318086, 27904015	< 1kb
25757625, 25897126	1.2 kb
27210041	2.2 kb
27234594	4 kb
23997119	10 kb
25137067	23 kb
25803037, 26742453	65 kb
26620761	95 kb
27396308	0.5 Mb

** 17 out of 36 refs (including PMID 23643243, 23929337, 24284873, 24315440, 25666713, 25997509, 26053263, 26216318, 26537785, 26817415, 26876963, 27223891, 27224051, 27258160, 27387532, 27483347, 27515175, 27530713, and 27685656) provided the information of large deletion generated by multiple sgRNA injection.

Supplementary Table 2. Summary of embryo microinjection of sgRNAs and Cas9 mRNA

1. Targeting individual genomic sites with single sgRNAs

Targeting strategies	Targeted genomic sites	sgRNA	Embryos injected	Pups born	Frequency of mutations (%)
One-step	B	1	36	18	44
		2	176	55	31
	C-1	1	150	14	79
		2	93	13	62
	D-2	1	150	57	84
	D-3	1	101	31	39
	D-4 (1)	1	27	43	53
	F	1	191	40	25
Two-step	C-2/3	1 (targeting site 2 in site C-3 mutants)	68	20	60

2. Targeting individual genomic sites with more than one sgRNA

Targeting strategies	Targeted genomic sites	sgRNA	Embryos injected	Pups born	Frequency of mutations (%)
One-step	A	1, 2	183	46	33
	C-1/3	1, 2	15	6	100
	D-4 (2)	1, 2	84	17	100
	E1	1, 2	75	75	9
	E2	1, 2	50	50	14
	E3	1, 2, 3	41	41	41
	E4	1, 2, 3	32	12	92
	E5	1, 2, 3	80	30	100
Two-step	D-1/2/3/4	1, 2 (targeting site 2 in site C-1/3/4 mutants)	38	4	50

3. Targeting more than one genomic site with several sgRNAs

Targeting strategies	Targeted genomic sites	sgRNA	Embryos injected	Pups born	Frequency of mutations (%)
One-step	D-1/3/4	1, 2, 3, 4	78	27	7
Two-step	C-1/2, C-1/2/3	1, 2, 3, 4 (targeting site 2 and 3 in site C-1 mutants)	90	23	91
	D-1/2/3/4/5	1, 2, 3, 4 (targeting site 2 and 5 in site D-1/3/4 mutants)	27	10	100

Supplementary Table 3. Frequency of insertions found in each targeted site

1. Targeting individual genomic sites with single sgRNAs

Targeting strategies	Targeted genomic sites	sgRNA	Insertion	Total founders	Insertion frequency (%)
One-step	B	1	0	18	0
		2	1	55	2
	C-1	1	1	14	7
		2	0	13	0
	D-2	1	12	57	21
	D-3	1	0	31	0
	D-4 (1)	1	2	43	5
	F	1	0	40	0
Two-step	C-2/3	1 (targeting site 2 in site C-3 mutants)	0	20	0
	Average				4

2. Targeting individual genomic sites with more than one sgRNA

Targeting strategies	Targeted genomic sites	sgRNA	Insertion	Total founders	Insertion frequency (%)
One-step	A	1, 2	4	46	9
	C-1/3	1, 2	4	6	67
	D-4 (2)	1, 2	0	17	0
	E-1	1, 2	1	75	1
	E-2	1, 2	0	50	0
	E-3	1, 2, 3	0	41	0
	E-4	1, 2, 3	1	12	8
	E-5	1, 2, 3	1	30	3
Two-step	D-1/2/3/4	1, 2 (targeting site 2 in site C-1/3/4 mutants)	0	4	0
	Average				10

3. Targeting more than one genomic site with several sgRNAs

Targeting strategies	Targeted genomic sites	sgRNA	Insertion	Total founders	Insertion frequency (%)
One-step	D-1/3/4	1, 2, 3, 4	0	27	0
Two-step	C-1/2, C-1/2/3	1, 2, 3, 4 (targeting site 2 and 3 in site C-1 mutants)	4	23	17
	D-1/2/3/4/5	1, 2, 3, 4 (targeting site 2 and 5 in site D-1/3/4 mutants)	0	10	0
	Average				6

Supplementary Table 4. Frequency of misleading genotypes linked to large deletions

Targeted genomic sites	Mosaic large deletions (%)	Founders w/ deletion	Founders w/ mosaic large deletions	Number of alleles	Deletion size (bp)
C-1/2/3	10	20	2	2<br>3	10/ 338(w/ +800 bp)<br>6/ 446/ 2,300
D-1/2/3/4/5	33	9	3	2<br>3<br>4	0/ 23,339<br>240/ 22,284/ 23,987<br>118/ 22,147/ 23,287/ 23,520
D-1/2/3/4	100	1	1	2	2,504/ 15,506

Supplementary Note 1. Targeting individual genomic sites with single sgRNAs.

a) Genomic site B (A STAT5 binding site in *Socs2* promoter)

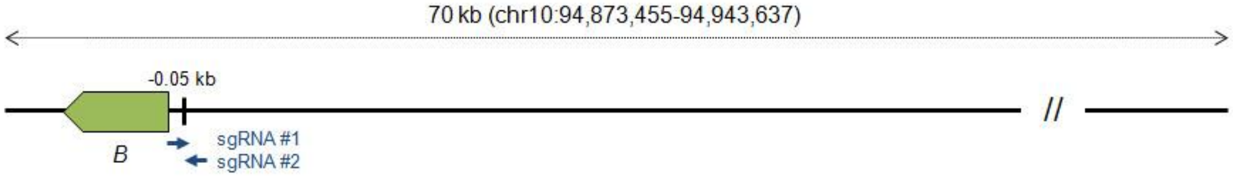

1) sgRNA sequence

sgRNA	sgRNA sequence	Co-injection (Yes/No)
sgRNA #1	5'-CCGCTTTCCAGGAACTTTCC-3'	No
sgRNA #2	5'-GGTCACGTGAGGCGGATTCC-3'	

2) Target site

- Blue: Target sequence (GAS motif)
- Yellow: Target NGG sequence complemented to sgRNA
- **Bold**: sgRNA #1 binding site
- Underline: sgRNA #2 binding site

GCAAGAGTTAACCGTCTGTCCCCGGAGCCGAGTGTGCGCGCGACTTCCCAGCCCGACGCGCCTCCCTCGCTCCCCGC
CTCCCGCCCCTCGCGCCCCGCCCGGCCGCGGCGGAGG**CCGCTTTCCAGGAACTTTCC**AGGAATCCGCCTCACGTGAC
CGCAGCCCCCGCCAGCGCTTTAAAGAGGCCCCGGCCGCTAATGCGCAGGCTTCAAGGGAGCCGGGTGGAGAGAGCCG
GTGCGGGTGCTGGTGCTGGTGGCTGCGGGGTGGAAGCCTGCGACTCCGCCCTCCTCTGACCCTGCACCTCGGGGTGC
TCGGGAACAAGGAGAAGTCCCCAGCCAATGCCTATTAAGCCACCAGTACCTGCCCCTGTGTCTGCAACAGCTG
AGCACCTCGGTCCCGCGAATAGCAGCCCCTCTTC

3) Mutated sequence

- Blue: Target sequence (GAS motif)
- Yellow: Target NGG sequence complemented to sgRNA
- … red dotted line: Cas9 cutting site (between 3rd and 4th nucleotide upstream of NGG site)
- --- : deletion sequence, red: insertion or point mutation
- +1: putative Cas9 cutting site (3 nucleotide upstream of target NGG sequence)
- - bp: deletion size, + bp: insertion size, -: unknown (only sequenced from one end)
- A: asymmetric deletion, S: symmetric deletion, 5': 5'-deletion, 3': 3'-deletion
- Green: repeat sequence aligned at one end (or microhomology), Pink: repeat sequence aligned at both ends

No.	Sequence (w/ sgRNA #1)	Mutation start point	Mutation size (bp)	Direction of deletion
WT	GGCCGCT*TTCCAGGAA*CT*TTCCAGGAA*TCCGCCTCACGTGACCGCAG			
6995	GGCCGCT*TTCCAGGAA*---*TCCAGGAA*TCCGCCTCACGTGACCGCAG	-3	-3	A(5')
6997	CCCGG------…-------*TCCAGGAA*TCCGCCTCACGTGACCGCAG	-27	-27	A(5')
6998	GGCCGCT*TTCCAGGAA*---*TCCAGGAA*TCCGCCTCACGTGACCGCAG	-3	-3	A(5')
6999	GGCCGCT*TTCCAGGAA*--*TTCCAGGAA*TCCGCCTCACGTGACCGCAG	-3	-2	A(5')
7000	GGCCGCT*TTCCAGGAA*--*TTCCAGGAA*TCCGCCTCACGTGACCGCAG	-3	-2	A(5')
7005	GGCCGCT*TTCCAGGAA*CT----*AGGAA*TCCGCCTCACGTGACCGCAG	-1	-4	A(3')
7007	GGCCGCT*TTCCAGGAA*--*TTCCAGGAA*TCCGCCTCACGTGACCGCAG	-3	-2	A(5')
6980	CCCGG------…-------------------------…------CCGCA	-29	-51	S (29:22)

No.	Sequence (w/ sgRNA #2)	Mutation start point	Mutation size (bp)	Direction of deletion
WT	GAGGCCGCTTTCCAGGAACTTTCCAGGAATCCGCCTCACGTGACCGC			
93	GAGGCCGCTTTCCAGGAACTTT---------…---------CTTTA	-33	-39	A(5')
94	GAGGCCGCTTTCCAGGAACTTTCCAGGAA------------------	-	-	A(5')
95	GAGGCCGCTTTCCAGGAACTTTCCAGGA-------------------	-	-	A(5')
96	GAGGCCGCTTTCCAGGAACTTTCCAGGA-------------------	-	-	A(5')
124	GAGGCCGCTTTCCAGGAACTTTCCAGGAA------------------	-	-	A(5')
126	GAGGCCGCTTTCCAGGAACTTTCCAGGA-------------------	-	-	A(5')
127	GAGGCCGCTTTCCAGGAACTTTCCAGGA-------------------	-	-	A(5')
128	CCTCG------…-------------------------…-------CGCAG	-27	-64	S (37:27)
130	GAGGCCGCTTTCCAGGAACTTTCCAGGA-------------------	-	-	A(5')
131	GAGGCCGCTTTCCAGGAACTTTCCAGGAA--AGCCTCAC…TGACCGC	-2	-3, +1	A(5')
134	GCTCC------…-------------------------…-------CCCGC	-34	-88	A(3') (54:34)
137	GCCCC------…-------------------------…-------CGCAG	-27	-58	A(3') (42:16)
143	GAGGCCGCTTTCCAGGAACT---------TCCGCCTCACGTGACCGC	-1	-9	A(3')
145	CCCCG--…--TCCAGGAACTTTCC--------GCCTCACGTGACCGC	+18;<br>-4	-43;<br>-8	A(3')<br>S
147	GAGGCCGCTTTCCAGGAACTTTCC--------GCCTCACGTGACCGC	-4	-8	S
148	GAGGCCGCTTTCCAGGAACTTTCCAGGA-------------------	-	-	A(5')
149	CCCGG------…-------------------------…-------CTAAT	-54	-85	A(5') (31:54)

b) Genomic site C-1 (A STAT5 binding site in *Wap* enhancer)

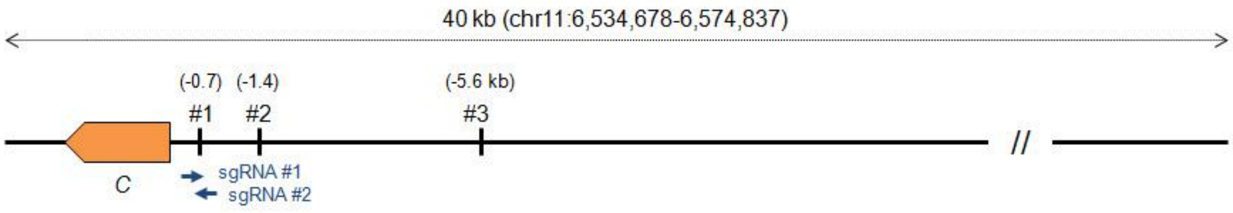

1) sgRNA sequence

Target site		sgRNA sequence	Co-injection (Yes/No)
C-1	sgRNA #1	5'-TGGAAGGCCACTTCCCAGAA-3'	No
	sgRNA #2	5'-GGCACAGTATGGGCCCTTCT-3'	

2) Target site

- Blue: Target sequence (GAS motif)
- Yellow: Target NGG sequence complemented to sgRNA
- **Bold**: sgRNA #1 binding site
- Underline: sgRNA #2 binding site

TTCTCCTGGTGCTTTTCTGGAGTTTTCTAAAAATTCTTCTAAGAGTGTGGAGGCGCTTGCTCCATGTAGAGAGTGTC
GGGCATACATTGAAAAGGAGCTGCCTGTGCAGAGCACAT**TGGAAGGCCACTTCCCAGAA**GGGCCCATACTGTGCCAA
GGGTGTCATGGGCAGGAAGAGATGTCCTGCTGGGCACTGTGCCCCATTGTTCTCGGGACATTTCCTTGATTTCTCTT
GCAAGGAAGGGGATGGGATGACCCAGAGTGACTGTGTGCTTACTGACTGTCAGCGCCAGCCTGTGGTGCAGGCCATA
CTCCTTTGTCTTCACTGGTGGCCCATGGCCTCTTTGAATACGAAGTGTCAGGGGAAGCCCTCTCAGGCTGTAACCTC
G

3) Mutated sequence

- Blue: Target sequence (GAS motif)
- Yellow: Target NGG sequence complemented to sgRNA
- … red dotted line: Cas9 cutting site (between 3rd and 4th nucleotide upstream of NGG site)
- --- : deletion sequence, red: insertion or point mutation
- +1: putative Cas9 cutting site (3 nucleotide upstream of target NGG sequence)
- - bp: deletion size, + bp: insertion size, -: unknown (only sequenced from one end)
- A: asymmetric deletion, S: symmetric deletion, 5': 5'-deletion, 3': 3'-deletion
- Green: repeat sequence aligned at one end (or microhomology), Pink: repeat sequence aligned at both ends

No.	Sequence (w/ sgRNA #1)	Mutation start point	Mutation size (bp)	Direction of deletion
WT	ATTGGAAGGCCACTTCCCAGAAGGGCCCATACTGTGCCAAGGGTGTC			
76	ATTGGAAGGCCACTTCCCAGAATGGGCCCATACTG…CCAAGGGTGTC	+4	+1	
77	ATTGGAAGGCCACTTCCCAGAAG------------------------	+5	-	A(3')
78	ATTGGAAGGCCACTTCCCAGAA-------------------------	+4	-	A(3')
80	ATTGGAAGGCCACTTCCCA----------------------------	+1	-	A(3')
81	ATTGGAAGG---------------GCCCATACTGTGCCAAGGGTGTC	-10	-15	A(5')
84	ATTGGAAGGCCACTTCCCAGAA-------------------------	+4	-	A(3')
85	ATTGGAAGGCCAC------------CCCATACTGTGCCAAGGGTGTC	-6	-12	S
106	ATTGGAAGGCCA-----------GGCCCATACTGTGCCAAGGGTGTC	-7	-11	A(5')
107	ATTGGAAGGCCACTTCCC-----------TACTGTGCCAAGGGTGTC	-1	-11	A(3')
108	ATTGGAAGGCCACTTCC-----------ATACTGTGCCAAGGGTGTC	-2	-11	A(3')
110	AT-------------------------CATACTGTGCCAAGGGTGTC	-17	-25	A(5')

No.	Sequence (w/ sgRNA #2)	Mutation start point	Mutation size (bp)	Direction of deletion
WT	AGAGCACATTGGAAGGCCACTTCCCAGAAGGGCCCATACTGTGCCAA			
87	AGAGCACATTGGAAGGCCA---------------CATACTGTGCCAA	-6	-15	A(3')
88	AGAGCACATTGGAAGGCCACTTCCCA----------TACTGTGCCAA	-8	-10	A(5')
91	AGAGCACATTGGAAGG-----------------CCATACTGTGCCAA	-5	-17	A(3')
112	AGAGCACATTGGAAGGCCA---------------------------A	-18	-27	A(5')
113	AGAGCACATTGGAAGGCCACTTC-------GGCCCATACTGTGCCAA	-2	-7	A(3')
115	AGAGCACATTGGAAGGCCACTTCCCAG-------------…-GGGAA	-224	-225	A(5')
116	TGCCT----------…---------------…----------TCTCT	-90	-122	A(5') (32:90)
118	AGAGCACATTGGAAGGCCACTTCCCAGA-------------------	-	-	A(5')

c) Genomic site C-2/3 (A STAT5 binding site in *Wap* enhancer)

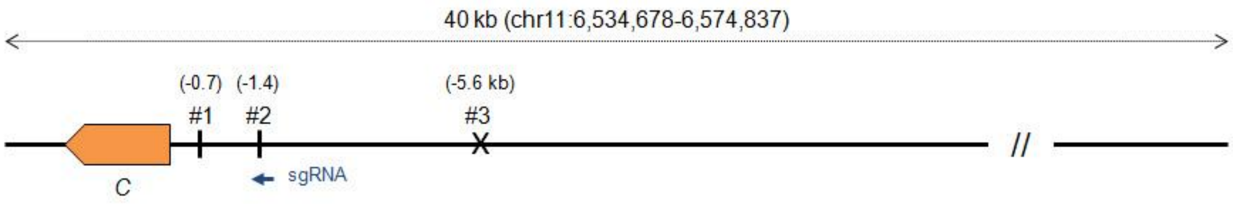

*** The sgRNA targeting site C-2 was injected into zygotes from mice carrying deletion of C-3.*

1) sgRNA sequence

Target site	sgRNA sequence	Co-injection (Yes/No)
C-2	5'-CCTAAGACACAGGGCCTTCT-3'	No

2) Target site

- Blue: Target sequence (GAS motif)
- Yellow: Target NGG sequence complemented to sgRNA
- Underline: sgRNA binding site

GGCTACTGCCATGCTTGTGTTAGTCAAGACTAAGTCATACCTATGCTCTGCCAATGCTCTGCTTCCACCCCTGTTCC
CATTTCCGCCTTTGCTGGAACAGAAGGAGGGCCACCAGGAAAGGGCTGGCTAGGGTTGCTTGGCTGCTTGAGTTTCC
CAGAAGGCCCTGTGTCTTAGGAGCCAGAGCTCTGTTCTCTCTGCATCTTGCTGGGGCCCATGCACTCTGTGTCCTAT
GCCCACTGGCTGTTATTCTTGCCTATGTACCA

3) Mutated sequence

- Blue: Target sequence (GAS motif)
- Yellow: Target NGG sequence complemented to sgRNA
- ... red dotted line: Cas9 cutting site (between 3rd and 4th nucleotide upstream of NGG site)
- --- : deletion sequence, red: insertion or point mutation
- +1: putative Cas9 cutting site (3 nucleotide upstream of target NGG sequence)
- - bp: deletion size, + bp: insertion size, -: unknown (only sequenced from one end)
- A: asymmetric deletion, S: symmetric deletion, 5': 5'-deletion, 3': 3'-deletion
- Green: repeat sequence aligned at one end (or microhomology), Pink: repeat sequence aligned at both ends

No.	Sequence (site C-2)	Mutation start point	Mutation size (bp)	Direction of deletion
WT	GGTTGCTTGGCTGCTTGAGTTTCCCAGAAGGCCCTGTGTCTTAGGAG			
1398	GGTTGCTTGGCTGCTTGAGTTTCCC---------TGTGTCTTAGGAG	-6	-9	A(5')
1400	GGTTGCTTGGCTGCTTGAGTTTCCC------CCCTGTGTCTTAGGAG	-3	-6	S
1401	GGTTGCTTGGCTGCTTGAGTTTCCCAG-AGGCCCTGTGTCTTAGGAG	+1	-1	A(3')
1403	GGTTGCTTGGCTGCTTGAGTTTCCCAG-AGGCCCTGTGTCTTAGGAG	+1	-1	A(3')
1404	GGTTGCTTGGCTGCTTGAGTTTCC--------CCTGTGTCTTAGGAG	-4	-8	S
1413	GGTTGCTTGGCTGCTTGAGTTTCCC------CCCTGTGTCTTAGGAG	-3	-6	S
1416	GGTTGCTTGGCTGCTTGAGTTTCCC---------TGTGTCTTAGGAG	-6	-9	A(5')
1417	GGTTGCTTGGCTGCTTGAGTTTCCCAGA-GGCCCTGTGTCTTAGGAG	-1	-1	A(5')
1418	GGTTGCTTGGCTGCTTGAGTTTCCCAGA---CCCTGTGTCTTAGGAG	-3	-7	A(5')
1420	GGTTGCTTGGCTGCTTGAGTTTCCC--------CTGTGTCTTAGGAG	-5	-8	A(5')
1421	GGTTGCTTGGCTGCTTGAGTTTCCC------CCCTGTGTCTTAGGAG	-3	-6	S
1422	GGTTGCTTGGCTGCTTGAGTTTCCC------CCCTGTGTCTTAGGAG	-3	-6	S

No.	Sequence (site C-3)	Mutation start point	Mutation size (bp)
WT	GGTCGGGAAGCCAGAAGGTTCTAGGAATGAAGACATTACTCTGAATT		
All	GGTCGGGAAGCCAGAA-----------TGAAGACATTACTCTGAATT	-6	-11

d) Genomic site D-2 (A CTCF binding site in *Wap* locus)

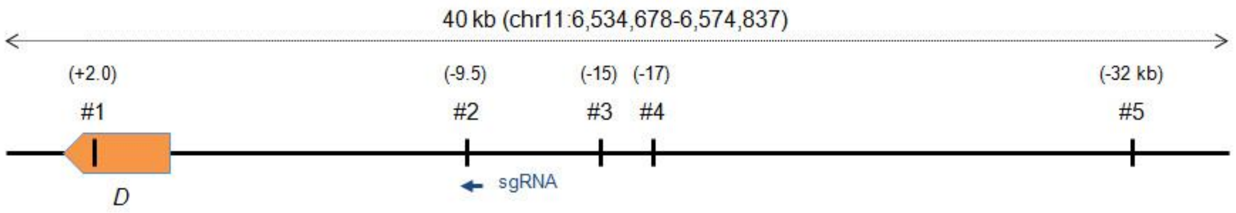

1) sgRNA sequence

Target site	sgRNA sequence	Co-injection (Yes/No)
D-2	5’-TAACTCATATGGGCCAGTGG-3’	No

2) Target site

- Blue: Target sequence (CTCF binding site)
- Yellow: Target NGG sequence complemented to sgRNA
- Underline: sgRNA binding site

GCTGGATAGCTCTTGCCAGGTGTTTCTTCTGCTGAGAATGATGAGCGGCCCTGACACCTCAAAGCCGCTTTGCTCCT
TTGCACATGGATCTCAGAAAAAGCCTCTGAGTTTCCCATATGAACTCAGGAGACCCAACCCAACCCAACCCAACCCA
ACCCAACCCAACCCAACCCAACCCAACAAACACCCAAACCGAGAGAAATAACAAAACGGCTCACAGCTCCCTCCACT
GGCCCATATGAGTTACTGCAGCTGCTTTATCAAAAAACAGCTTCTGGGTCCACAGCAGAGCCAGGCTTTCTTCCAAC
CTTTCTAGGTCAAAGCCAAGTCCTGAGCTCCCAGTTCTTTTCCTTGCTTTCTCCACGATACATGTCATCCCTGTTGA
CCCAGCTTGTTCAGCCACACCGGTTTCCTGATGGCCCTCTCCTGCAGGAACCTC

3) Mutated sequence

- Blue: Target sequence (CTCF binding site)
- Yellow: Target NGG sequence complemented to sgRNA
- … red dot line: Cas9 cutting site (between 3rd and 4th nucleotide upstream of NGG site)
- --- : deletion sequence, red: insertion or point mutation
- +1: putative Cas9 cutting site (3 nucleotide upstream of target NGG sequence)
- - bp: deletion size, + bp: insertion size, -: unknown (only sequenced from one end)
- A: asymmetric deletion, S: symmetric deletion, 5’: 5’-deletion, 3’: 3’-deletion
- Green: repeat sequence aligned at one end (or microhomology), Pink: repeat sequence aligned at both ends

No.	Sequence	Mutation start point	Mutation size (bp)	Direction of deletion
WT	ACAAAACGGCTCACAGCTCCCTCCACTGGCCCATATGAGTTACTGCA			
9011	ACAAAACGGCTCACAGCTCCCTCCACCTGGCCCATATGAGTTACTGC	+2	+1	
9012	ACAAAACGGCTCACAGCTCCCTCCA-----CCATATGAGTTACTGCA	-2	-5	A(3’)
9013	ACAAAACGGCTCACAGCT------------CCATATGAGTTACTGCA	-2	-12	A(3’)
9016	ACAAAACGGCTCACAGCTC------CTGGCCCATATGAGTTACTGCA	+4	-6	A(3’)
9018	ACAAAACGGCTCACAGCTCCCTCCA-TATGGCCCATATGAGTTACTG	+1	-1, +2	A(3’)
9019	ACAAAACGGCTCACAGCTCCCTCCA--------TATGAGTTACTGCA	-5	-8	A(5’)
9020	ACAAAACGGCTCACAGCTCCCTCCA-----CCATATGAGTTACTGCA	-2	-5	A(3’)
9021	ACAAAACGGCTCACAGCTCCCTCCATCTGGCCCATATGAGTTACTGC	+3	+1	
9022	ACAAAACGGCTCACAGCTCCCTCCACCTGGCCCATATGAGTTACTGC	+3	+1	

9024	ACAAAACGGCTCACAGCTCCCTCCA--------TATGAGTTACTGCA	-5	-8	A(5')
9025	ACAAAACGGCTCACAGCTCCCTCC------CCATATGAGTTACTGCA	-2	-6	A(3')
9026	ACAAAACGGC-------------------GGCCCATATGAGTTACTGCA	+2	-17	A(3')
9027	CCAAC-----…------------------CCATATGAGTTACTGCA	-2	-68	A(3')
9028	ACAAAACGGCTCACAGCTCCCTCCAC----CCATATGAGTTACTGCA	-2	-4	S
9031	ACAAAACGGCTCACAGCTCCCTCCA--------TATGAGTTACTGCA	-5	-8	A(5')
9033	ACAAAACGGCTCACAGCTCCCTCCA--------TATGAGTTACTGCA	-5	-8	A(5')
9034	ACAAAACGGCTCACAGCTCCCTCCA---------ATGAGTTACTGCA	-6	-9	A(5')
9035	ACAAAACGGCTCACAGCTCCCTCC-CTGGCCCATATGAGTTACTGCA	+4	-1	A(3')
9037	ACAAAACGGCTCACAGCTCCCTCCATCTGGCCCATATGAGTTACTGC	+3	+1	
9039	ACAAAAC-----------------------------…------AAAAC	-32	-53	A(5') (21:32)
9040	ACAAAACGGCTCACAGCTCCCTCCATCT--GGCCCATATGAGTTACT	-2	+1, -2	A(5')
9041	ACAAAACGGCTCACAGCTCCCTCCA--------TATGAGTTACTGCA	-5	-8	A(5')
9042	ACAAAACGGCTCACAGCTCCCTCCA-TGGCCCATATGAGTTACTGCA	+3	-1	A(3')
9044	ACAAAACGGCTCACAGCTCCCTC---TGGCCCATATGAGTTACTGCA	+3	-3	A(3')
9045	ACAAAACGGCTCACAGCTCCCTCC------CATATGAGTTACTGCA	-3	-7	S
9046	CCAAC-----…----------------------TGAGTTACTGCA	-7	-73	A(3')
9047	CCAAC-----…----------------------…------AGCTC	-70	-177	S (76:101)
9048	ACAAAACGGCTCACAGCTCCCTCCA----CCATATGAGTTACTGCA	-2	-5	A(3')
9049	ACAAAACGGCTCACAGCTCCCTCC-CTGGCCCATATGAGTTACTGCA	+4	-1	A(3')
9050	ACAAAACGGCTCACAGCTCCCTCCATCTGGCCCATATGAGTTACTGC	+3	+1	
9052	ACA--------------------------CCATATGAGTTACTGCA	-2	-27	A(3')
9053	ACAAAACGGCTCACAGCTCCC-----------ATATGAGTTACTGCA	-4	-11	A(3')
9054	ACAAAACGGCTCACAGCTCCC---------CCATATGAGTTACTGCA	-2	-9	A(3')
9056	ACAAAACGGCTCACAGCTCCCTCC------CCATATGAGTTACTGCA	-2	-6	A(3')
9139	ACAAAACGGCTCACAGCTCCCTCC--------ATATGAGTTACTGCA	-4	-8	S
9140	ACAAAACGGCTCACAGCTCCCTCC------CCATATGAGTTACTGCA	-2	-6	A(3')
9141	ACAAAACGGCTCACAGCTCCCTCCACATGGCCCATATGAGTTACTGC	+2	+1	
9142	ACAAAACGGCTCACAGCTCCCTCCA----CCATATGAGTTACTGCA	-2	-5	A(3')
9143	ACAAAACGGCTCACAGCTCCCTC-----…--------…------TCTGG	-41	-46	A(5')
9144	ACAAAACGGCTCACAGCTCCCTCCATA-TGGCCCATATGAGTTACTG	+1	+2, -1	A(3')
9145	ACAAAACGGCTCACAGCTCCCTCCATA-TGGCCCATATGAGTTACTG	+1	+2, -1	A(3')
9146	ACAAAACGGCTCACAGCTCCCTCC--------ATATGAGTTACTGCA	-4	-8	S
9147	ACAAAACGGCTCACAGCTCCCTCC--------ATATGAGTTACTGCA	-4	-8	S
9149	ACAAAACGGCTCACAGCTCCCTCCATCTGGCCCATATGAGTTACTGC	+3	+1	
9150	ACAAAACGGCTCACAGCTCCC---------CCATATGAGTTACTGCA	-2	-9	A(3')
9151	ACAAAACGGCTCACAGCTCCCTCCAACTGGCCCATATGAGTTACTGC	+3	+1	
9152	ACAAAACGGCTCACAGCT----------------------…------ACAGC	-35	-45	A(5')
9153	ACAAAACGGCTCACAGCTCCCTCCA----CCATATGAGTTACTGCA	-2	-5	A(3')

e) Genomic site D-3 (A CTCF binding site in *Wap* locus)

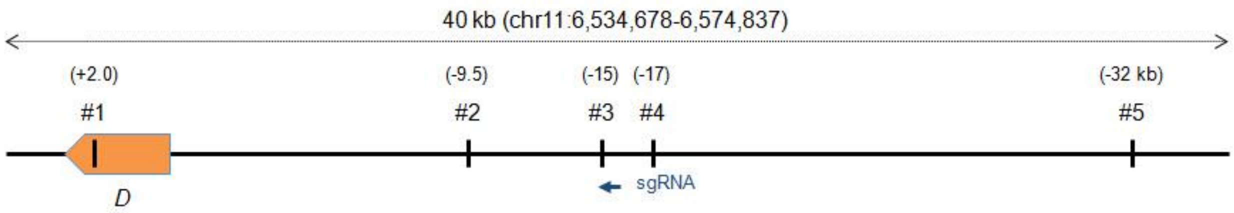

1) sgRNA sequence

Target site	sgRNA sequence	Co-injection (Yes/No)
D-3	5'-CTTCTGGTGGGCACAGGTAC-3'	No

2) Target site

- Blue: Target region (CTCF binding site)
- Yellow: Target NGG sequence complemented to sgRNA
- Underline: sgRNA binding site

CCGGCCCAGGTTTGTTTTTCAGGACCCTCTTGGTGGCTCACAACTTTCTGGAACTCATAGTTGCAAGGGGATCTGAC
ATCCTCTTCTGGTGGGCACAGGTACTGCATGCATGTGGTACACTGTCATACATGTAGACAAATCAACCAGATACATA
AGTTAGAAATGAAGAAACTGGGCTGGTGAGATGGCTCAGTGGGTAAGAGTACCCGACTGCTCTTC

3) Mutated sequence

- Blue: Target sequence (GAS motif)
- Yellow: Target NGG sequence complemented to sgRNA
- ... red dotted line: Cas9 cutting site (between 3rd and 4th nucleotide upstream of NGG site)
- --- : deletion sequence, red: insertion or point mutation
- +1: putative Cas9 cutting site (3 nucleotide upstream of target NGG sequence)
- - bp: deletion size, + bp: insertion size, -: unknown (only sequenced from one end)
- A: asymmetric deletion, S: symmetric deletion, 5': 5'-deletion, 3': 3'-deletion
- Green: repeat sequence aligned at one end (or microhomology), Pink: repeat sequence aligned at both ends

No.	Sequence	Mutation start point	Mutation size (bp)	Direction of deletion
WT	TAGTTGCAAGGGGATCTGACATCCTCTTCTGGTGGGCACAGGTACTG			
1257	TAGTTGCAAGGGGATCTGACAT--------...----------GAGATG	-96	-102	A(5')
1260	TAGTTGCAAGGGGATCTGAC----------...----------GAAACT	-82	-90	A(5')
1263	TAGTTGCAAGGGGATCT----------------------...-GGGCTG	-82	-93	A(5')
1269	TAGTTGCAAGGGGATCTGACA--------------------GACAAA	-52	-59	A(5')
1275	TAGTTGCAAGGGGATCTG---------------GGGCACAGGTACTG	-5	-15	A(3')
1277	TAGTTGCAAGGGGATCTGACAT--------GGTGGGCACAGGTACTG	-2	-8	A(3')
1278	TAGTTGCAAGGGGATCTGAC----------...----------GACTGC	-123	-131	A(5')
1283	TAGTTGCAAGGGGATCTGACAT-------------------AACCAC	-178	-184	A(5')
1284	CCCTCCTCCT--...---------------------GGCACAGGTACTG	-6	-258	A(3')
1285	TAGTTGCAAGGGGATCTGACATCCTCTT--...----------AACTAA	-295	-295	A(5')
1286	TAGTTGCAAGGGGATCTGACATC-------...----------ATGAAG	-281	-286	A(5')
1289	TAGTTGCAAGGGGATCTGACA---------...----------TACAGT	-229	-236	A(5')

f) Genomic site D-4 (1) (A CTCF binding site in *Wap* locus)

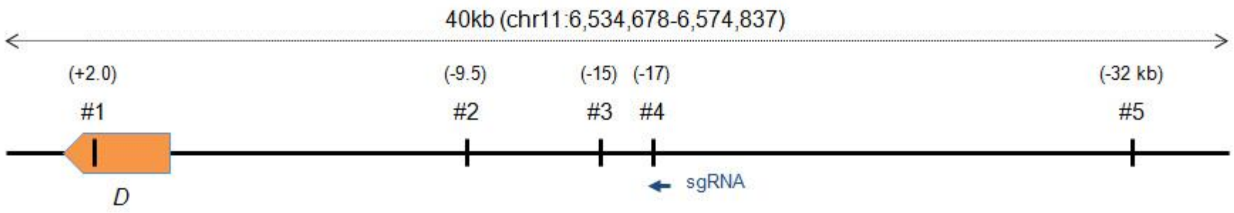

1) sgRNA sequence

Target site	sgRNA sequence	Co-injection (Yes/No)
D-4	5'-AACTCAGTCTGTGGTAGATG-3'	No

2) Target site

- Blue: Target sequence (CTCF binding region)
- Yellow: Target NGG sequence complemented to sgRNA
- Underline: sgRNA binding site

CCCTGACTTGGTCATTATGCATTGTATTGAAGAGCCTCATTGGACCTTATAAATATGTATTATTACTATGTGTTAAT
TAAAATACATTTTAAAAAGAAGACAGAATTGGATAGTGAGTTGTAGGCTGGGTCAGAAACATTCCTTTATGTAATTC
TCCCTTCTGCAAGTATGTTAAGTCTACCTCATCTACCACAGACTGAGTTACTTAAATGTTATTCCAGCAGGGGGCGC
CAAATCATCAGGAATGCCTCTGAGGCAGTCTCGTTGGATGGGAGAAGAAACTAATCTCTGATGCTTCTTGAGTGTGT
GTGTGTGTGTGTGTGTGTGTGTGTGTATGTGTGATAGGTCTGGTTAGTATATCTGTGTGTGATTTGTGTATGTGTCT
CATATGTGTGTGGTTGTGTGTATCATGTGTAAATGTGTATGTGTATTGTATATGTGTGTACATATGTGCGTGCTGTA
TGTGCAACGTGCATATGTATGTGTGCACACGTGCTCGTG

3) Mutated sequence

- Yellow: Target NGG sequence complemented to sgRNA
- ... red dotted line: Cas9 cutting site (between 3rd and 4th nucleotide upstream of NGG site)
- --- : deletion sequence, red: insertion or point mutation
- +1: putative Cas9 cutting site (3 nucleotide upstream of target NGG sequence)
- - bp: deletion size, + bp: insertion size, -: unknown (only sequenced from one end)
- A: asymmetric deletion, S: symmetric deletion, 5': 5'-deletion, 3': 3'-deletion
- Green: repeat sequence aligned at one end (or microhomology), Pink: repeat sequence aligned at both ends

No.	Sequence	Mutation start point	Mutation size (bp)	Direction of deletion
WT	*TTCTGCAAGTATGTTAAGTCTACCTCATCTACCACAGACTGAGTTAC*			
9011	*TTCTGCAAGTATGTTAAGTCTACC---------ACAGACTGAGTTAC*	-5	-9	S
9012	*TTCTGCAAGTATGTTAAGTCTACC---------ACAGACTGAGTTAC*	-5	-9	S
9013	*TTCTGCAAGTATGTTAAGTCTACCTCAT-------------------*	-	-	A(5')
9016	*TTCTGCAAGTATGTTAAGTCTACCTCAT--ACCACAGACTGAGTTAC*	-2	-2	A(5')
9023	*TTCTGCAAGTATGTTAAGTCTACCTCATTCTACCACAGACTGAGTTA*	-1	+1	
9033	*TTCTGCAAGTATGTTAAGTCTACCTCATCT-----------------*	-	-	A(5')
9034	*TTCTGCAAGTATGTTAAGTCTACC---------ACAGACTGAGTTAC*	-5	-9	S
9035	*TTCTGCAAGTATGTTAAGTCTACCTCAT-------------------*	-	-	A(5')
9036	*TTCTGCAAGTATGTTAAGTCTACCTCA-CTACCACAGACTGAGTTAC*	+1	-1	A(3')

9040	TTCTGCAAGTATGTTAAGTCTACC---------ACAGACTGAGTTAC	-5	-9	S
9041	TTCTGCAAGTATGTTAAGTCTACC---------ACAGACTGAGTTAC	-5	-9	S
9042	TTCTGCAAGTATGTTAAGTCTACC---------ACAGACTGAGTTAC	-5	-9	S
9044	TTCTGCAAGTATGTTAAGTCTACCTCAT-------------------	-	-	A(5')
9049	TTCTGCAAGTATGTTAAGTCTACC---------ACAGACTGAGTTAC	-5	-9	S
9051	TTCTGCAAGTATGTTAAGTCTACCTCAT-------------------	-	-	A(5')
9053	TTCTGCAAGTATGTTAAGTCTACCTCATCTTACCACAGACTGAGTTA	-3	+1	
9054	TTCTGCAAGTATGTTAAGTCTACC---------ACAGACTGAGTTAC	-5	-9	S
9056	TTCTGCAAGTATGTTAAGTCTACC---------ACAGACTGAGTTAC	-5	-9	S
9139	TTCTGCAAGTATGTTAAGTCTACC---------ACAGACTGAGTTAC	-5	-9	S
9140	TTCTGCAAGTATGTTAAGTCTACC----------CAGACTGAGTTAC	-6	-10	A(5')
9143	TTCTGCAAGTATGTTAAGTCTACC----------CAGACTGAGTTAC	-6	-10	A(5')
9145	TTCTGCAAGTATGTTAAGTCTACCT---------CAGACTGAGTTAC	-6	-9	A(5')
9147	TTCTGCAAGTATGTTAAGTCTACC---------ACAGACTGAGTTAC	-5	-9	S

g) Genomic site F (An NFIB binding site in *Csn3* enhancer)

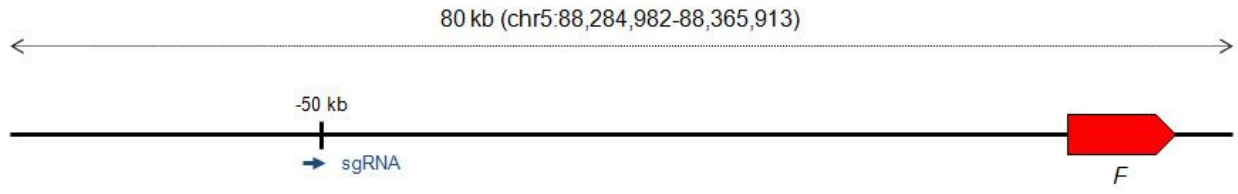

1) sgRNA sequence

sgRNA sequence	Co-injection (Yes/No)
5'- AACAACCTAACTCTTGAAAT-3'	No

2) Target site

- Blue: Target sequence (NFIB binding motif)
- Yellow: Target NGG sequence complemented to sgRNA
- Underline: sgRNA binding site

CTACATGCTTAGCGTTTGTTCATTTAAGAAAATGATTAAAGATTAATTTTCAGTGATGGGCAGCAGTTATTCATAAT
CAAAGCCCTGATGGTAAAAGCCTGCTGAGCTGTAATAAATGACCTATAAACTGAAGCCGTGTCCTTCTGAGGAGCTC
TCTGGAATCATTGATGTGTAGATAACATAGATGGAGGGGGAAAAAAAAACAACAACCTAACTCTTGAAATTGGCAAG
CTATCTAAATAACTGGGACAACTGCAGTTCAGTTTTGCCTCTGGGTACACAGGTGGCTGGCCAGTGGCCAGTCCTTT
TCAGCTCTGGCTGGCAATGTCATTGTCCTGAGGGAATTCAGGAAGGCAGTGGCTTCGGTTGGGTTAGATGGACTTCT

3) Mutated sequence

- Blue: Target sequence (NFIB binding motif)
- Yellow: Target NGG sequence complemented to sgRNA
- … red dot line: Cas9 cutting site (between 3rd and 4th nucleotide upstream of NGG site)
- --- : deletion sequence, red: insertion or point mutation
- +1: putative Cas9 cutting site (3 nucleotide upstream of target NGG sequence)
- - bp: deletion size, + bp: insertion size, -: unknown (only sequenced from one end)
- A: asymmetric deletion, S: symmetric deletion, 5': 5'-deletion, 3': 3'-deletion
- Green: repeat sequence aligned at one end (or microhomology), Pink: repeat sequence aligned at both ends

No.	Sequence	Mutation start point	Mutation size (bp)	Direction of deletion
WT	CAACAACCTAACTCTTGAAAT*TGGCA*AGCTATCTAAATAACTGGGAC			
1	TCCTT-…--------------------GCTATCTAAATAACTGGGAC	-78	-87	A(5’)
2	CAACAACCTAACTCTT------*GGCA*AGCTATCTAAATAACTGGGAC	-2	-6	A(3’)
4	CAACAACCT----------------------------…-ATACTAAA	-9	-449	A(3’)
13	CAACAACCTAACTCT----------------------…-GGGTATAT	-3	-385	A(3’)
14	CAACAACCTAACT------------------------…-TGTCATTG	-5	-109	A(3’)
18	CAACAA---------------------GCTATCTAAATAACTGGGAC	-12	-21	S (12:9)
20	CAACAACCTAACTCTTGA-------------------…-CTTCTCAC	+1	-159	A(3’)
21	CAA----------------------------------…-GCTTTATA	-15	-585	A(3’)
26	CAACAACCTAACTCTT---------------------…-GTGGAAAT	-2	-364	A(3’)
31	CAACAACCTAACTCT----------------------…-GTTCTCTC	-3	-199	A(3’)

Supplementary Note 2. Targeting individual genomic sites with more than one sgRNA.
a) Genomic site A (A STAT5 binding site in *Stat5* enhancer)

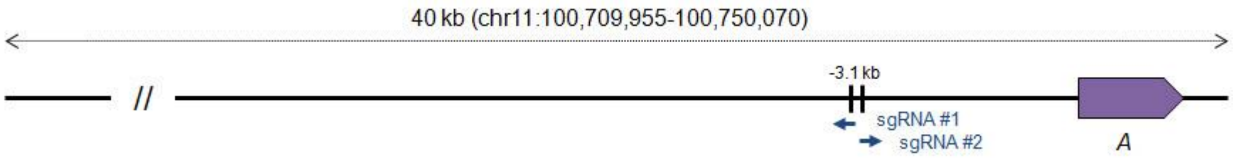

1) sgRNA sequence

sgRNA	sgRNA sequence	Co-injection (Yes/No)
sgRNA #1	5'-TCCTTTGTTTCTGGGAATAG-3'	Yes
sgRNA #2	5'-TGAAGAGTGTTAGAGACCAG-3'	

2) Target site

- Blue: Target sequence (GAS motif)
- Yellow: Target NGG sequence complemented to sgRNA
- **Bold**: sgRNA #1 binding site
- Underline: sgRNA #2 binding site

TGTGTGCCTAGAGTACCTGACCTGCTGGTCTCTCATCTGGGTAGGTGGGCATGGCAGCTGGCAAAGAAAGCAAGGTT
CCTAGCCTCCACTGCACCACCCAGGCTAGTCCCACAGCCTCCCTCCCATC**CCTCTATTCCCAGAAACAAAGGA**CAGT
CACTGTGCCTCCGGCAGGGTAGCCTTGTGTTCTCTTGGTGAGTTCTCTGTAAATTGACTTCTCTGAAGAGTGTTAGA
GATGTTCCAGGGGACAGAGATGGAAAGGGGGCTTCACCCCAATCCCAAGGCTCCTTACCCTTCTCTGCATTTACAGA
GAACACACACACACACACACACACACACACACACACACACACACACACATTCCCCGACTCTGGCCTATTCTGGCTTCAT
GATC

3) Mutated sequence

- Blue: Target sequence (GAS motif)
- Yellow: Target NGG sequence complemented to sgRNA
- --- : deletion sequence, red: insertion or point mutation
- +1: putative Cas9 cutting site (3 nucleotide upstream of the nearest target NGG sequence)
- - bp: deletion size, + bp: insertion size, -: unknown (only sequenced from one end)

No.	Sequence	Mutation start point	Mutation size (bp)
WT	sgRNA #1 66 bp 14 bp sgRNA #2<br>CATCCCTCTATTCCCAGAAAC…ACTTCTCTGAAGAG…CAGGGGACAG		
7001	CATCCCTCTATTCCCAGAAAC…ACTTCTCTGAA…AG-------ACAG	-1 (sgRNA #2)	-7
7009	CATCCCTCTATTCCCAGAAAC…ACTTCTCTGAAGAG…-AGGGGACAG	+1 (sgRNA #2)	-1
7011	CATCCCTCTATTCCCAGAAAC…ACTTCTCTGAA…GACCC…GGGACAG	-1 (sgRNA #2)	+1
7012	CATCCCTCTATTCCCAGAAAC…ACTTCTCTGAAGAG…-AGGGGACAG	+1 (sgRNA #2)	-1
7014	CATCCCTCTATTCCCAGAAAC…ACTTCTCTGAAGAG…-AGGGGACAG	+1 (sgRNA #2)	-1
7020	CATCCCTCTA---------AC…ACTTCTCTGAAGAG…CAGGGGACAG	-9 (sgRNA #1)	-9
7079	CATCCCTCTATTCCCAGAAAC…ACTTC---------…------…-AC	-19 (sgRNA #2)	-47
7080	CATCCCTCTATTCCCAGAAAC…ACTTCTCTGAAGAGTG-…---ACAG	-8 (sgRNA #2)	-14
7082	CATCCCTCTATTTCCCAGAAA…ACTTCTCTGAAGAG…CAGGGGACAG	-3 (sgRNA #1)	+1
7090	CATCCCTCTATTCCCAGAAAC…ACTTCTC…AG-TGTTCC…GGGACAG	-5 (sgRNA #2)	-1, +4
7094	CATCCCTCTATTCCCAGAAAC…ACTTCTCT…AGA----AGGGGACAG	-3 (sgRNA #2)	-4
7098	CATCCC---------AGAAAC…ACTTCTCTGAAGAG…CAGGGGACAG	-5 (sgRNA #1)	-9

7103	CATCCCTCTA*TTCCCAGAA*AC…AC*TTCTCTGAA*G…AC-AGAGGACAG	+1 (sgRNA #2)	-1, +1
7104	CATCCCTCTA*TTCCCAGAA*AC…AC*TTCTCTGAA*GA…GA-…---…-TG	-4 (sgRNA #2)	-14
7106	CATCCC----------*AGAA*AC…AC*TTCTCTGAA*GAG…CAGGGGACAG	-5 (sgRNA #1)	-9

b) Genomic site C-1/3 (STAT5 binding sites in *Wap* enhancer)

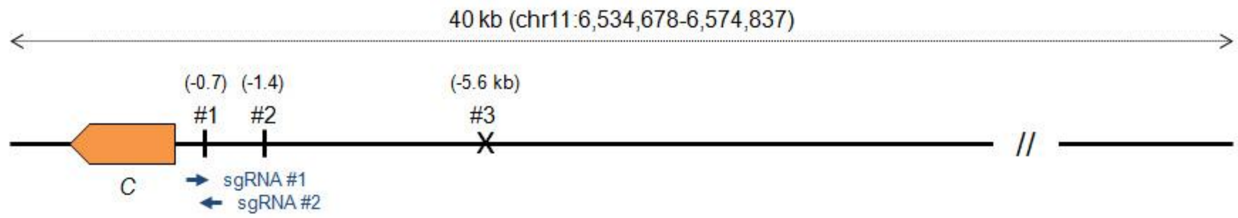

*** sgRNAs targeting C-1 were injected into zygotes from mice carrying deletion of C-3.*

1) sgRNA sequence

Target site		sgRNA sequence	Co-injection (Yes/No)
C-1	sgRNA #1	5'-TGGAAGGCCACTTCCCAGAA-3'	Yes
	sgRNA #2	5'-GGCACAGTATGGGCCCTTCT-3'	

2) Target site

- Blue: Target sequence (GAS motif)
- Yellow: Target NGG sequence complemented to sgRNA
- **Bold**: sgRNA #1 binding site
- Underline: sgRNA #2 binding site

TTCTCCTGGTGCTTTTCTGGAGTTTTCTAAAAATTCTTCTAAGAGTGTGGAGGCGCTTGCTCCATGTAGAGAGTGTC
GGGCATACATTGAAAAGGAGCTGCCTGTGCAGAGCACAT**TGGAAGGCCACTTCCCAGAA**GGGCCCATACTGTGCCAA
GGGTGTCATGGGCAGGAAGAGATGTCCTGCTGGGCACTGTGCCCCATTGTTCTCGGGACATTTCCTTGATTTCTCTT
GCAAGGAAGGGGATGGGATGACCCAGAGTGACTGTGTGCTTACTGACTGTCAGCGCCAGCCTGTGGTGCAGGCCATA
CTCCTTTGTCTTCACTGGTGGCCCATGGCCTCTTTGAATACGAAGTGTCAGGGGAAGCCCTCTCAGGCTGTAACCTC
G

3) Mutated sequence

- Blue: Target sequence (GAS motif)
- Yellow: Target NGG sequence complemented to sgRNA
- --- : deletion sequence, red: insertion or point mutation
- +1: putative Cas9 cutting site (3 nucleotide upstream of the nearest target NGG sequence)
- - bp: deletion size, + bp: insertion size, -: unknown (only sequenced from one end)

No.	Sequence (site C-1)	Mutation start point	Mutation size (bp)
WT	*sgRNA #1* *sgRNA #2*<br>CACATTGGAAGGCCAC*TTCCCAGAA*GGGCCCATACTGTGCCAAGGGT		
3325	CACATTGGAAGGCCAC*TTCCCAAAA*GGGCCCATACTGTGCCAAGGGT	+1 (sgRNA #2)	-1, +1
3326A	CACATTGGAA-----------------GGCCCATACTGTGCCAAGGGT	+4 (sgRNA #2)	-16
3326B	CACATTGGAAGG----------------GCCCATACTGTGCCAAGGGT	+5 (sgRNA #2)	-15
3326C	CACATTGGAAGGCCAC*TTCCCAAG*…GGGCCCATACTGTGCCAAGGGT	-1 (sgRNA #2)	+1

3327A	CACATTGGAAGGCCA--------------CCATACTGTGCCAAGGGT	-5 (sgRNA #1)	-14
3327B	CACATTGGAAGGCCAC*TTCCCAAGA*GGGCCCATACTGTGCCAAGGGT	+1 (sgRNA #2)	+2
3328A	CACATTGGAA----------------GGCCCATACTGTGCCAAGGGT	+4 (sgRNA #2)	-16
3328B	CACATTGGAAGG---------------GCCCATACTGTGCCAA-GGT	+12 (sgRNA #1)<br>+22 (sgRNA #1)	-15<br>-1
3329A	CACATTGGAAGGCCA--------------CCATACTGTGCCAAGGGT	+7 (sgRNA #2)	-14
3329B	CACATTGGAAGGCCAC*TTCCCAGA*-GGGCCCATACTGTGCCAAGGGT	+3 (sgRNA #2)	-1
3330	CACATTGGAAGGCCAC*TTCCCAAG*…GGGCCCATACTGTGCCAAGGGT	-1 (sgRNA #2)	+1

No.	Sequence (site C-3)	Mutation start point	Mutation size (bp)
WT	GGTCGGGAAGCCAGAAGG*TTCTAGGAA*TGAAGACATTACTCTGAATT		
All	GGTCGGGAAGCCAGAA-----------TGAAGACATTACTCTGAATT	-6	-11

c) Genomic site D-4 (2) (A CTCF binding site in *Wap* locus)

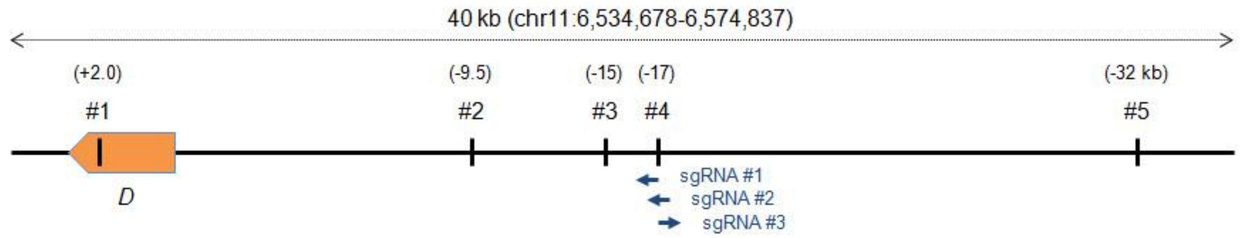

1) sgRNA sequence

Target site		sgRNA sequence	Co-injection (Yes/No)
D-4	sgRNA #1	5'-GACTTAACATACTTGCAGAA-3'	Yes
	sgRNA #2	5'-AACTCAGTCTGTGGTAGATG-3'	
	sgRNA #3	5'-CTGAGGCAGTCTCGTTGGAT-3'	

2) Target site

- Blue: Target sequence (CTCF binding region)
- Yellow: Target NGG sequence complemented to sgRNA
- **Bold**: sgRNA #1 binding site
- Underline: sgRNA #2 binding site
- **Bold** and Underline: sgRNA #3 binding site

ACATTTTAAAAAGAAGACAGAATTGGATAGTGAGTTGTAGGCTGGGTCAGAAACATTCCTTTATGTAATTCTCCC**TT**
CTGCAAGTATGTTAAGTCTACCTCATCTACCACAGACTGAGTTACTTAAATGTTATTCCAGCAGGGGGCGCCAAATC
ATCAGGAATGCCT**CTGAGGCAGTCTCGTTGGAT**GGGAGAAGAAACTAATCTCTGATGCTTCTTGAGTGTGTGTGTGT
GTGTGTGTGTGTGTGTGTGTATGTGTGATAGGTCTGGTTAGTAT

3) Mutated sequence

- Yellow: Target NGG sequence complemented to sgRNA
- --- : deletion sequence, red: insertion or point mutation
- +1: putative Cas9 cutting site (3 nucleotide upstream of the nearest target NGG sequence)
- - bp: deletion size, + bp: insertion size, -: unknown (only sequenced from one end)
-

No.	Sequence	Mutation start point	Mutation size (bp)
WT	sgRNA #1 11 bp sgRNA #2 78 bp sgRNA #3<br>GTAATTCTCCCTTCTG…AGTCTACCTCATCTAC…ATGGGAGAAGAAACT		
1114	ATTT-…----------…----------------…----------…TCTG	+20 (sgRNA#1)	-146
1115	ATTC-…----------…----------------…----------…TCTC	+20 (sgRNA#1)	-144
1126	GTAATTCTCCC-----…----------------…----------…TGTG	+8 (sgRNA#1)	-160
1127	CATT-…----------…----------------…----------…CTCT	+21 (sgRNA#1)	-146
1116	GTAA------------…----------------…------GAAGAAACT	+10 (sgRNA#1)	-123
1120	GTAATTCTCCCT----…----------------…ATGGGAGAAGAAACT	+2 (sgRNA#1)	-107
1121	GTAATTCTCCC…TAT-…----------------…---------GAAACT	-10 (sgRNA#1)	-107
1124	GTAATTCTCCC-----…----------------…-TGGGAGAAGAAACT	+3 (sgRNA#1)	-111
1137	GTAATTCTCCCTT---…----------------…--GGGAGAAGAAACT	+1 (sgRNA#1)	-110
1128	GTAATTCTCCCTTCT-…------------CTAC…ATGGGAGAAGAAACT	-2 (sgRNA#1)	-24
1131	GTAATTCTCCCTTCT-…------------CTAC…ATGGGAGAAGAAACT	-2 (sgRNA#1)	-24
1122	GTAA------------…-GTCTACCTCATCTAC…ATGGGAGAAGAAACT	+10 (sgRNA#1)	-24
1117	GTAATTCTCCCTTCTG…AGTCTACCT…CT-…-GGATGGGAGAAGAAACT	-3 (sgRNA#2)	-78
1132	GTAATTCTCCCTTCT-…-AGTC…CCTCATCTAC…ATGGGAGAAGAAACT	-2 (sgRNA#1)	-12
1119	GTAATTCTCCCTTCTG…AGTCTACCTCA-…-GA…ATGGGAGAAGAAACT	+1 (sgRNA#2)	-9
1133	GTAATTCTCCCTTCT-…-TATG…CCTCATCTAC…ATGGGAGAAGAAACT	-2 (sgRNA#1)	-5
1118	GTAATTCTCCCTTCTG…AGTCTACCTCATC-AC…ATGGGAGAAGAAACT	-2 (sgRNA#2)	-1

d) Genomic site D-1/2/3/4 (CTCF binding sites in *Wap* locus)

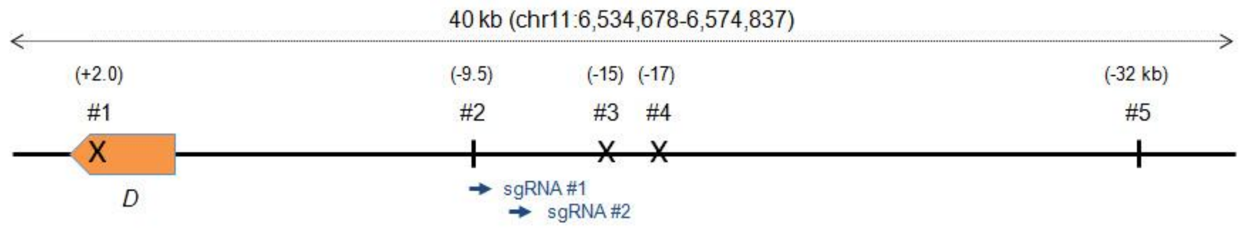

*** sgRNAs targeting site D-2 were injected into zygotes from mice carrying deletion of D-1/3/4.*

1) sgRNA sequence

Target site		sgRNA sequence	Co-injection (Yes/No)
D-2	sgRNA #1	5'-TGAGTTTCCCATATGAACTC-3'	Yes
	sgRNA #2	5'-TCCTGATGGCCCTCTCCTGC-3'	

2) Target site

- Blue: Target sequence (CTCF binding region)
- Yellow: Target NGG sequence complemented to sgRNA
- **Bold**: sgRNA #1 binding site
- Underline: sgRNA #2 binding site

GGATAGCTCTTGCCAGGTGTTTCTTCTGCTGAGAATGATGAGCGGCCCTGACACCTCAAAGCCGCTTTGCTCCTTTG
CACATGGATCTCAGAAAAAGCCTC**TGAGTTTCCCATATGAACTC**AGGAGACCCAACCCAACCCAACCCAACCCAACC

CAACCCAACCCAACCCAACCCAACAAACACCCAAACCGAGAGAAATAACAAAACGGCTCACAGCTCCCTCCACTGGC
CCATATGAGTTACTGCAGCTGCTTTATCAAAAAACAGCTTCTGGGTCCACAGCAGAGCCAGGCTTTCTTCCAACCTT
TCTAGGTCAAAGCCAAGTCCTGAGCTCCCAGTTCTTTTCCTTGCTTTCTCCACGATACATGTCATCCCTGTTGACCC
AGCTTGTTCAGCCACACCGGTTTCCTGATGGCCCTCTCCTGCAGGAACCTCTCGCTGAGGTCAAGTGACTACA

3) Mutated sequence

- Yellow: Target NGG sequence complemented to sgRNA
- --- : deletion sequence, red: insertion or point mutation
- +1: putative Cas9 cutting site (3 nucleotide upstream of target NGG sequence)
- - bp: deletion size, + bp: insertion size, -: unknown (only sequenced from one end)

No.	Sequence	Mutation start point	Mutation size (bp)
WT	*sgRNA #1 283 bp sgRNA #2*<br>*ATATGAACTCAGGAGACCCAACC…CCTCTCCTGCAGGAACCTCTCGC*		
2392A	AAG--…-------------…--------------------…--ATA	-1,627	-2,504
2392B	AGC--…-------------…--------------------…--GTG	-1,084	-15,506

e) Genomic site E-1 (A CTCF binding site in *Csn1s1* locus)

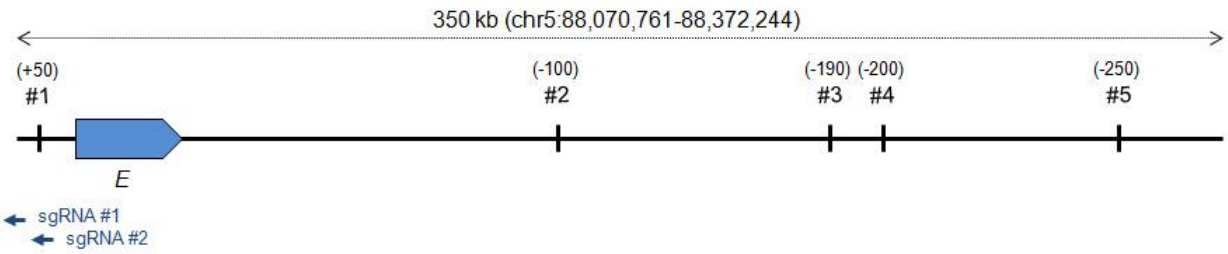

1) sgRNA sequence

Target site		sgRNA sequence	Co-injection (Yes/No)
E-1	sgRNA #1	5'-CAGTTCAGTGGTTAATCAAG-3'	Yes
	sgRNA #2	5'-ACACTAATTTTGACCAGTAG-3'	

2) Target site

- Blue: Target sequence (CTCF binding site)
- Yellow: Target NGG sequence complemented to sgRNA
- **Bold**: sgRNA #1 binding site
- Underline: sgRNA #2 binding site

CCTGTTGCTGGACCTTCTTTTCCTCAGGCTCCTCTCCATTTCCATCCCTGTAATTCTTTCAGACAGGAACATTATGG
GTCAGAATTTTGATAGTTCACAATTTTATGCAAAGAGGTGAGCATTGTGGAGCTTGCCACTTGATTAACCACTGAAC
TGGACATGCAAGATGTGGCTGCCCTCTACTGGTCAAAATTAGTGTTCACAGCTGGATGGTTCAGTCCCTTTTTTGGG
GGGGGGGGGGTTGAAATTAGACATTTTTTAAATTTACATTTCAAATTTTATCCCCTTTTCTGGTTTCCCCCATATC
CCTGCTCCCTCCACCTGCCTCACTGCCCTAGCATTCCCCTATATTAGAGCATTGAGCCTCCACAAAACCAAGGGCTT
CTCTTCC

3) Mutated sequence

- Blue: Target sequence (CTCF binding motif)
- Yellow: Target NGG sequence complemented to sgRNA
- --- : deletion sequence, red: insertion or point mutation
- +1: putative Cas9 cutting site (3 nucleotide upstream of the nearest target NGG sequence)
- - bp: deletion size, + bp: insertion size, -: unknown (only sequenced from one end)

No.	Sequence	Mutation start point	Mutation size (bp)
WT	sgRNA #1 20 bp sgRNA #2<br>AGCTTGCCACTTGATTAAC…ATGTGGCTGCCCTCTACTGGTCAAAAT		
8960	AGC…G…CCACTTGATTAAC…ATGTGGCTGCCCTCTACTGGTCAAAAT	+10 (sgRNA#1)	+10
8966	GTGGA----------TTAA…ATGTGGCTGCCCTC---…---AAAATT	+11 (sgRNA#1)<br>+2 (sgRNA#2)	-13<br>-8
8968	AGCTTGCCACT-…-GAACT…ATGTGGCTGCCC-----…----TCACAG	+1 (sgRNA#1)<br>+4 (sgRNA#2)	-12<br>-21
8969	AGCTTG------…--CCAC…ATGTGGCTGCCCTCTACTGGTCAAAAT	+6 (sgRNA#1)	-12
8970	CACA-…------…---TGG…ATGTGGCTGCCCTCT---…--AAAATT	+39 (sgRNA#1)<br>+1 (sgRNA#2)	-54<br>-7
8972	AGCTTGCCACTTGATTAAC…ATGTGGCTGCCCTCT---GGTCAAAAT	+1 (sgRNA#2)	-3
8973	AGCTTGCCACTTGA-…-ACTGAA…GGCTGCCC-----…----TTTTTG	-3 (sgRNA#1)<br>+4 (sgRNA#2)	-6<br>-44

f) Genomic site E-2 (A CTCF binding site in *Csn1s1* locus)

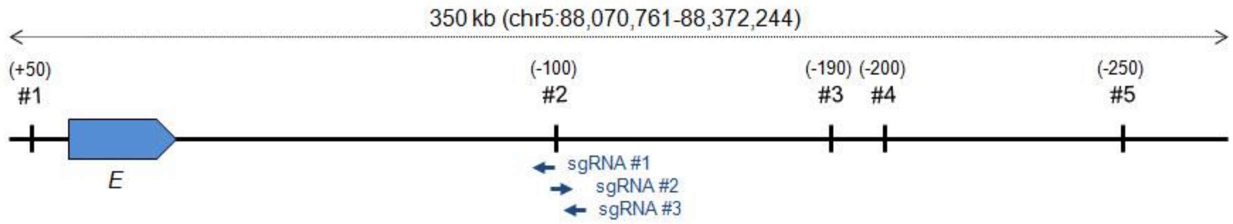

1) sgRNA sequence

Target site		sgRNA sequence	Co-injection (Yes/No)
E-2	sgRNA #1	5'-GATACAGATTTTAAGTGCCT-3'	Yes
	sgRNA #2	5'-GTTTTATTTTGTCCAGGAGA-3'	
	sgRNA #3	5'-TTATTTGATTGCTGGCTAAG-3'	

2) Target site

- Blue: Target sequence (CTCF binding region)
- Yellow: Target NGG sequence complemented to sgRNA
- **Bold**: sgRNA #1 binding site
- Underline: sgRNA #2 binding site
- **Bold** and Underline: sgRNA #3 binding site

CTTTTCCACTGAGACCCAACCATACATTCCAAGTAGGAGGGAATGGGATCCAATGGCAGTGAACAGAGACTGAGTCA
GCCGCTGTTGCACTTGTTAGGGGAGTTATATGAACATTAAACTACGTATTTGCTACAGTTGTGTACAGGGTCTAGGC
CCAGATCCTGCATGTTTTCTGGTTGGTGGTGTCCT**AGGCACTTAAAATCTGTATC**TGAAGTAGAAACGTTTTAGTGT

TTTATTTTGTCCAGGAGATGGCAGAGTTGTGATGCAAATACTGTACATTAGTTAGTAAATCTCACCTC**TTAGCCAGC**
AATCAAATAATAGGCCTTTTTGCCCCCCCCCCCACAGCAGAACAAGTTATTTAATGTATAACTCCTTGCAATAGTAA
CCTTTCATTTTGAGGATTTCGTTTATGAGCAATGAAGTTGCATCAATCTGATCCCCCTCTTTCCTCTCCCACCTCAA
ATCCAAGACTGTTTATTCTTTAATCAATTGTTTCATAGAAAGCATATACAGGCATGTATGTATGTATGTATGTATGT

3) Mutated sequence

- Yellow: Target NGG sequence complemented to sgRNA
- --- : deletion sequence, red: insertion or point mutation
- +1: putative Cas9 cutting site (3 nucleotide upstream of the nearest target NGG sequence)
- - bp: deletion size, + bp: insertion size, -: unknown (only sequenced from one end)

No.	Sequence	Start point	Deletion size (bp)
WT	sgRNA #1 48 bp sgRNA #2 31 bp sgRNA #3<br>TGGTGTCCTAGGCAC…AGGAGATGGCAGAGT…ATCTCACCTCTTAGC		
467	TGGTGTCCTAGG---…---AGATGGCAGAGT…ATCTCACCTCTTAGC	-1 (sgRNA#1)	-54
469	GTCTA----…----TCTG…AGA-------GT…ATCTCACCTCTTAGC	+41 (sgRNA#1)<br>+4 (sgRNA#2)	-56<br>-7
470	TGGTGTCCTAGGCA--…----------…TGATG…TCTCACCTCTTAGC	-3 (sgRNA#1)	-66
480	TGGTGTC---…---TGTA…AGATGGCAGAGT…GTTA-…---…-TTTT	+5 (sgRNA#1)<br>+16 (sgRNA#3)	-16<br>-40
485	TGGTGTCC-----…------------------…-----------…-TTGC	+4 (sgRNA#1)	-139
488	TGGTGTCC-----…------------------…-----------…-CTCT	+4 (sgRNA#1)	-253
489	TGGTGTCCTA---…------------------…-----------…-TTGC	+2 (sgRNA#1)	-232

g) Line E-3 (A CTCF binding site in *Csn1s1* locus)

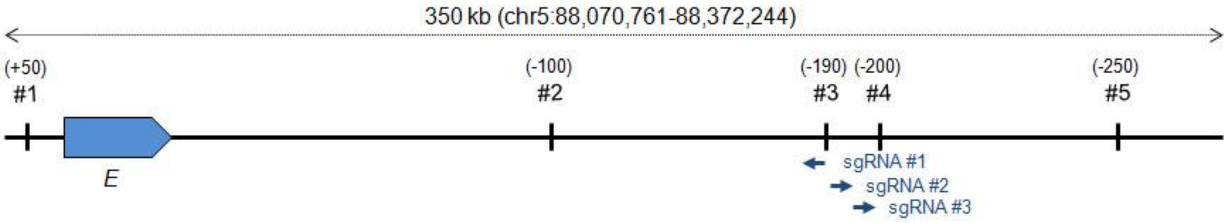

1) sgRNA sequence

Target site		sgRNA sequence	Co-injection (Yes/No)
#3	sgRNA #1	5'-ACAAATTGTCTGCTGAAGGC-3'	Yes
	sgRNA #2	5'-CCTCCTCTAGAGCGGAAACA-3'	
	sgRNA #3	5'-AAATATTGTTCGGTGCCTCA-3'	

2) Target site

- Blue: Target sequence (CTCF binding site)
- Yellow: Target NGG sequence complemented to sgRNA
- **Bold**: sgRNA #1 binding site
- Underline: sgRNA #2 binding site
- **Bold** and Underline: sgRNA #3 binding site

CAGATGGCGATGTTTCTGGCTACGTACCCAGAAACCTTCTCCAGACCCGAAATTACAGAGGCTGTCAAGACACTGGT
CTCAATAGTAACACACATTTCTCATTTGCCTGTTTGATCCTTCAAATTTAAGTGACTCTAACCATTATGATGACGAT
TCTTTCAAGCACCTGCCTTCAGCAGACAATTTGTTGGACCTTCCTGTAGGTGGTAGTGAAGGGCTTCTAGATTTTGC
ACCTCCTCTAGAGCGGAAACAAGGTAATTTCAGGTGCTAGAAAATATTGTTCGGTGCCTCAAGGTCAGCACTTACTA
GCAGGATAGACTGAGCTAACTGTTGTTTCTGGGTATCATTTTGGTCCCATAC

3) Mutated sequence

- Blue: Target sequence (CTCF binding region)
- Yellow: Target NGG sequence complemented to sgRNA
- --- : deletion sequence, red: insertion or point mutation
- +1: putative Cas9 cutting site (3 nucleotide upstream of the nearest target NGG sequence)
- - bp: deletion size, + bp: insertion size, -: unknown (only sequenced from one end)

No.	Sequence	Start point	Deletion size (bp)
WT	sgRNA #1 72 bp sgRNA #2 25 bp sgRNA #3<br>CAAGCACCTGCCTTC…GAAACAAGGTAATTT…GCCTCAAGGTCAGCA		
9427	GCTG-…---------…---------------…---TCAAGGTCAGCA	+107 (sgRNA#1)	-225
9428	TTAA-…---------…---------------…---------…-CTTA	+43 (sgRNA#1)	-123
9429	CAAGCACCT…CAGCA…---------------…---------…-CTTA	-8 (sgRNA#1)	-123
9430	CAAGCACCTGCCTTC…GTAGT…---------…---------…-CTTA	-38 (sgRNA#2)	-90
9432	CAAGCACCTGCCTTC…GAA------------…--CTCAAGGTCAGCA	+1 (sgRNA#2)	-39
9433	CAAGCACCT…TAGA-…---------------…---------…-GGTA	-24 (sgRNA#2)	-156
9434	C-------------…-TTTTG…AGGTAATTT…GCCTCAAGGTCAGCA	+11 (sgRNA#1)	-65
9435	CAAGCACCT…AGCA-…---------------…---------…-CTTA	-8 (sgRNA#1)	-123
9436	CAAGCACCTGCCTTC…CCTC-…---------…-----AAAGTCAGCA	-10 (sgRNA#2)	-52
9482	CAAGCACCTGCCTTC…GCAC-…---------…---------…-TTAC	-16 (sgRNA#2)	-69
9483	CAAGCACCTGC…AGCG----CAAGGTAATTT…GCCTCAAGGTCAGCA	-3 (sgRNA#2)	-4
9484	CAAGCACCT…AGCAG------…---------…---------…-AAAT	-9 (sgRNA#1)	-195
9485	CAAGCACCTG-…-TTTGT…ACAAGGTAATTT…GCCTCAAGGTCAGCA	+2 (sgRNA#1)	-14
9486	CAAGCACCTG-----…---------TAATTT…GCCTCAAGGTCAGCA	+2 (sgRNA#1)	-86
9487	CAAGCACCTGCCTTC…GAA-CAAGGTAATTT…GCCTCAAGGTCAGCA	+1 (sgRNA#2)	-1
9489	CAAGCACCTGCCTTC-…-----------TTT…GCCTCAAGGTCAGCA	-4 (sgRNA#1)	-84
9503	CAAGCACCTGCC…GCGGA-----GGTAA…TCGGT-…------CAGCA	-1 (sgRNA#2)<br>-3 (sgRNA#3)	-5<br>-10

h) Line E-4 (A CTCF binding site in *Csn1s1* locus)

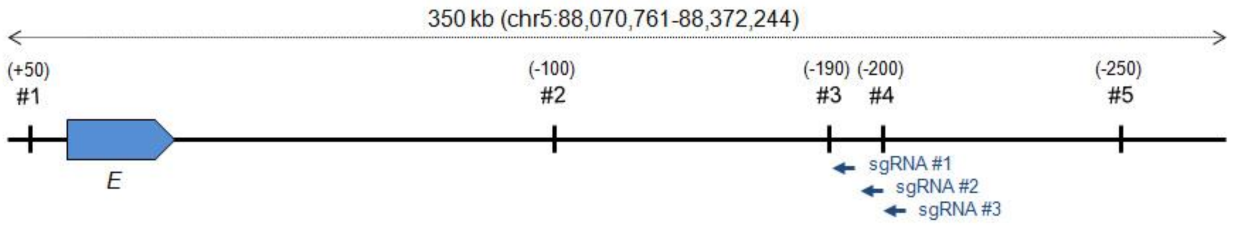

1) sgRNA sequence

Target site		sgRNA sequence	Co-injection (Yes/No)
#4	sgRNA #1	5'-AGGGCGCTGTTTGCCACAA-3'	Yes
	sgRNA #2	5'-TCTCTGAGCACTACCACACG-3'	
	sgRNA #3	5'-GGCACAGTGAGTCCCATTTG-3'	

2) Target site

- Blue: Target sequence (CTCF binding site)
- Yellow: Target NGG sequence complemented to sgRNA
- **Bold**: sgRNA #1 binding site
- Underline: sgRNA #2 binding site
- **Bold** and Underline: sgRNA #3 binding site

```
GTGCCTGGCAAACACAGAAGTGGATGCTCACAGTCAGCTACTGGATGGAACATAGGGCCCCCAATGGAGGAGCTAGA
GAAAGTACCCAAGGAGCTAAAGGGGTCTGCAACCCTATTGGTGGAACAACAATATGAAGTTTGCATTTTTATAAGGG
GAATAAAACAAAACAAAACAAAAAAAAACAAACAAACAAACAAAAAGACCATTGTGGCAAACAGCGCCCTCGTGTGGT
AGTGCTCAGAGACGTGCATCACTAATCAACAACCTTGGAGCTGTTGAAGGAGCTGATACTTAGCCAGGTTTGAGGGT
GACAGCTAAGCAGCACCCTCAAATGGGACTCACTGTGCCCATTTCGGTCACAAAAACTGACTTAGGTCCTTAAAATG
CATATCAGTTCAACATTCTAAATTGCTGCACACAGTCACTCAGTAGAGGCTCTCATGTATGATAAAATTCTACTTCT
TTTCTTTTTCTTTTTTTTCTTTTTTTGAGGTATATACATTTATCATTTCTTTTTTTTTCTTCTTTTCAATTAGCAATGT
```

3) Mutated sequence

- Blue: Target sequence (CTCF binding site)
- Yellow: Target NGG sequence complemented to sgRNA
- --- : deletion sequence, red: insertion or point mutation
- +1: putative Cas9 cutting site (3 nucleotide upstream of the nearest target NGG sequence)
- - bp: deletion size, + bp: insertion size, -: unknown (only sequenced from one end)

No.	Sequence	Start point	Deletion size (bp)
WT	*sgRNA #1* *4 bp* *sgRNA #2* *89 bp* *sgRNA #3*<br>AAAGACCATTGTGG…CAGCGCCCTCGTGTG…CAGCACCCTCAAATGG		
9490	AAAGACCATT----…---------------…----------…-TATTC	+1 (sgRNA#1)	-381
9491	AAAGACCATTG---…---------------…----------…-GGACT	-1 (sgRNA#1)	-126
9492	AAAGACC-T…TTGCAA…GCGCCCTCGTA…G-…-C…A----…-CTGTG	+4 (sgRNA#1)<br>-3 (sgRNA#1)<br>-1 (sgRNA#2)<br>-4 (sgRNA#2)<br>+7 (sgRNA#3)	-6<br>+5<br>+1<br>-6<br>-17
9494	AAAGACCATTGTGGC-…---------------…----------…-ACAAA	-5 (sgRNA#1)	-146
9495	AAAGACCATTG---…---------------…----------…-AAAAT	-1 (sgRNA#1)	-241
9496	AAAGACCATT----…---------------…----------…-ATTCA	+1 (sgRNA#1)	-382
9497	AAAGA---------…---------------…----------…-CCATT	+6 (sgRNA#1)	-145
9498	AAAGACC-------…----------…-TAGTG…---------CAAATGG	+4 (sgRNA#1)	-27
9499	AAAGACCATT----…---------------…----------…-TCGGT	+1 (sgRNA#1)	-145
9500	AAAGAC--------…---------------…----------…-ATGCA	+5 (sgRNA#1)	-180
9501	AAAGACCATTG---…---------------…----------…-AGGCT	-1 (sgRNA#1)	-224

i) Line E-5 (A CTCF binding site in *Csn1s1* locus)

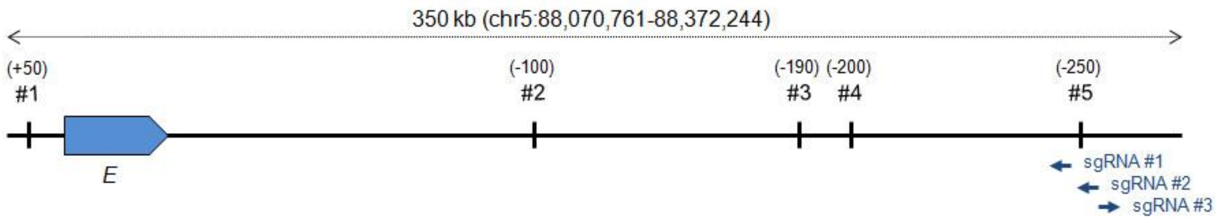

1) sgRNA sequence

Target site		sgRNA sequence	Co-injection (Yes/No)
#5	sgRNA #1	5'-TCCAGACTGGAACTGATCAA-3'	Yes
	sgRNA #2	5'-ATGCTCAAAATTACCTAAAG-3'	
	sgRNA #3	5'-GCTCTGATTGCTTGAATTGA-3'	

2) Target site

- Blue: Target sequence (CTCF binding site)
- Yellow: Target NGG sequence complemented to sgRNA
- **Bold**: sgRNA #1 binding site
- Underline: sgRNA #2 binding site
- **Bold** and Underline: sgRNA #3 binding site

GACAGTATTTATAGCCTGAGGAATCCAAGGCAATATTTAGCAGGGAAGATGTGAAAAGGGTAATGTTTGAAGTATAA
ATGGAATTTTCACTTTCTGGTGTGAGCCGTGTAACTAGTCACCAATTTGCATACCGTTTTAATATCTGAATTATATT
TTGTTTCCTTTGATCAGTTCCAGTCTGGAGAAAGTCCTAAGTCCAAGAGCTCCCTCTTTAGGTAATTTTGAGCATAA
AGATGTCTGGAGCTCTGATTGCTTGAATTGAAGGTACCAAACTGTCATGTTACTTTAACATAGATTACAGAGACATG
AAAGTATAAAAATCACAGTGACATCGTAAATAATACATGATAATCATGAATAAGAAGACAAGGAAGAAAACTAAACA
TACATGGAATATTGCTTTAACACGATTAATGAGAAATTAGAAACAATCTGTTAACTAGAAATTTGTCATTTAAAATA

3) Mutated sequence

- Blue: Target sequence (CTCF binding motif)
- Yellow: Target NGG sequence complemented to sgRNA
- --- : deletion sequence, red: insertion or point mutation
- +1: putative Cas9 cutting site (3 nucleotide upstream of the nearest target NGG sequence)
- \- bp: deletion size, + bp: insertion size, -: unknown (only sequenced from one end)

No.	Sequence	Start point	Deletion size (bp)
WT	*sgRNA #1* *31 bp* *sgRNA #2* *41 bp* *sgRNA #3*<br>TTGTTTCCTTTGATC…GAGCTCCCTCTTTAG…AATTGAAGGTACCAA		
9517	TTGTTTCCTTTG---…----------------…--TTGAAGGTACCAA	-1 (sgRNA#1)	-92
9519	TTGTT-----…-CCAGT…CTCCCTCTTT…AAAATTGAAGGTACCAA	+7 (sgRNA#1)<br>-2 (sgRNA#3)	-14<br>+2
9520	TTGTTTCCTTTG---…----------------…--TTGAAGGTACCAA	-1 (sgRNA#1)	-92
9521	TTGTTTCCTTTG---…----------------…--TTGAAGGTACCAA	-1 (sgRNA#1)	-92
9522	TTGTTTCCTTTG---…----------------…--TTGAAGGTACCAA	-2 (sgRNA#1)	-92
9523	TTGTTTCCTTTGATCA-…-------------…----GAAGGTACCAA	-5 (sgRNA#1)	-90
9525	TTGTTTCC-------…----------------…---------…-TGTC	+4 (sgRNA#1)	-111

9528	TTG-…----------…---------------…---------…-AAAC	+9 (sgRNA#1)	-112
9529	TTGTTT---------…---------------…---------…-TGTT	+6 (sgRNA#1)	-118
9530	TTGTTTCCTTT----…---------------…---------…-TGTT	+1 (sgRNA#1)	-113
9531	TATT-…---------…---------------…---------…-ACTG	+12 (sgRNA#1)	-117
9532	TTGTTTCCTTTG---…---------------…---------…-GTTA	-1 (sgRNA#1)	-113
9533	TTGTTTCCTTTG---…---------------…--TTGAAGGTACCAA	-2 (sgRNA#1)	-92
9534	TTGTTTCCTTTG---…---------------…---------…-AGAG	-1 (sgRNA#1)	-133
9535	TTGTTTCCTTT----…---------------…---------…-ACTG	+1 (sgRNA#1)	-106
9536	TTGTTTCCTTTG---…---------------…---------…-AGAG	-1 (sgRNA#1)	-133
9538	TTGTTTCCTTTG---…---------------…---TGAAGGTACCAA	-1 (sgRNA#1)	-93
9539	TTG------------…---------------…---------…-TGTT	+9 (sgRNA#1)	-121
9540	TTGTTTCCTTTG---…---------------…---------…-TACT	-1 (sgRNA#1)	-115
9541	TTGTTTCCTTTG---…---------------…----GAAGGTACCAA	-1 (sgRNA#1)	-94
9542	TTGTTTCCTTTG---…---------------…--TTGAAGGTACCAA	-2 (sgRNA#1)	-91
9543	TTGTTTCCTTTG---…---------------…---------…-ATAA	-2 (sgRNA#1)	-146
9544	TTGTTTCCTTTG---…---------------…-----AAGGTACCAA	-1 (sgRNA#1)	-95
9545	TTGT-----------…---------------…---------…-CATT	+8 (sgRNA#1)	-293
9546	TTGTTTCCTTTG---…---------------…---TGAAGGTACCAA	-1 (sgRNA#1)	-93
9547	TTGT-----------…---------------…---------…-GAAT	+8 (sgRNA#1)	-379
9548	TTGTTTCCTTTG---…---------------…--TTGAAGGTACCAA	-2 (sgRNA#1)	-91
9549	TATT-…---------…---------------…---------…-GCTT	+12 (sgRNA#1)	-244
9550	TTGTTTCC-------…---------------…--TTGAAGGTACCAA	+4 (sgRNA#1)	-96
9551	TTGT-----------…---------------…---------…-CATT	+8 (sgRNA#1)	-293

Supplementary Note 3. Targeting more than one genomic site with several sgRNAs.

a) Genomic site C-1/2 and C-1/2/3 (STAT5 binding sites in *Wap* enhancer)

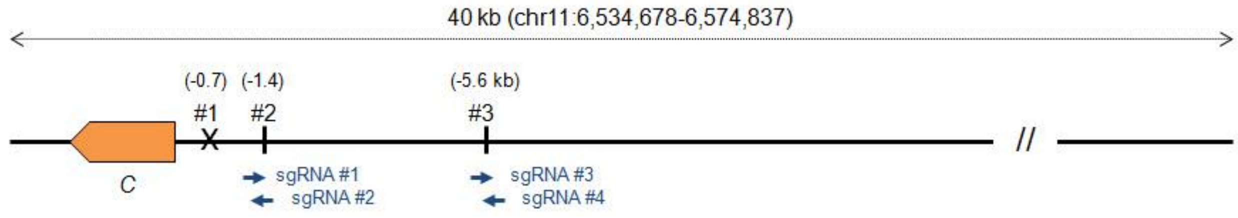

*** sgRNAs targeting site C-2 and C-3 were injected into zygotes from mice carrying deletion of C-1.*

1) sgRNA sequence

Target site		sgRNA sequence	Co-injection (Yes/No)
C-2	sgRNA #1	5'-GGCTGCTTGAGTTTCCCAGA-3'	Yes
	sgRNA #2	5'-CCTAAGACACAGGGCCTTCT-3'	
C-3	sgRNA #3	5'-TCGGGAAGCCAGAAGGTTCT-3'	Yes
	sgRNA #4	5'- TCTTCATTCCTAGAACCTTC-3'	

2) Target site

a. Site C-2

- Blue: Target sequence (GAS motif)
- Yellow: Target NGG sequence complemented to sgRNA
- **Bold**: sgRNA #1 binding site
- Underline: sgRNA #2 binding site

GGCTACTGCCATGCTTGTGTTAGTCAAGACTAAGTCATACCTATGCTCTGCCAATGCTCTGCTTCCACCCCTGTTCC
CATTTCCGCCTTTGCTGGAACAGAAGGAGGGCCACCAGGAAAGGGCTGGCTAGGGTTGCTT**GGCTGCTTGAGTTTCC**
CAGAAGGCCCTGTGTCTTAGGAGCCAGAGCTCTGTTCTCTCTGCATCTTGCTGGGGCCCATGCACTCTGTGTCCTAT
GCCCACTGGCTGTTATTCTTGCCTATGTACCA

b. Site C-3

- Blue: Target sequence (GAS motif)
- Yellow: Target NGG sequence complemented to sgRNA
- **Bold**: sgRNA #3 binding site
- Underline: sgRNA #4 binding site

ACTCAAGGGAACCTAGCCTTGAGGCCCAGGGTGACACTGGTCAGTTGAGGACATGTGTTCCTGTGTGGTGGGGAAGT
GTAGGGAGGACAGAGAGGCCCTGGCTGAACAAGGAGTTCATGAGGTGCACACATCTCCTCAGAGCCTGCAGGCCAGG
TCGGGAAGCCAGAAGGTTCTAGGAATGAAGACATTACTCTGAATTCACCAGGCCCAGCGGCAAGGGGCATGGGGTTC
TTATGCCCTGGGGTGTTCTGGTTCTTTGGCCTGCCAGTCTTGAGCCTGTGTGCTTCCAGTATGCGTAGGCTCTTTCC
TGCCCCATCAACT

3) Mutated sequence

- Blue: Target sequence (GAS motif)
- Yellow: Target NGG sequence complemented to sgRNA
- --- : deletion sequence, red: insertion or point mutation
- +1: putative Cas9 cutting site (3 nucleotide upstream of the nearest target NGG sequence)
- - bp: deletion size, + bp: insertion size, -: unknown (only sequenced from one end)

No.	Sequence (site C-1)	Mutation start point	Mutation size (bp)
WT	ATTGGAAGGCCAC*TTCCCAGAA*GGGCCCATACTGTGCCAAGGGTGTC		

All	ATTGGAAGGCC-----------------CATACTGTGCCAAGGGTGTC	-8	-16

No.	Sequence (site C-2 & C-3)	site	Mutation start point	Mutation size (bp)
WT	C-2 (sgRNA #1, #2) C-3 (sgRNA #3, #4)<br>GTTGCT…TTGAGTTTCCCAGAAGGCCC…CGGGAAGCCAGAAGGTTCTAGGAATGA			
251	GTTGC-…-----------------GCCC…CGGGAAGCCA----------AGGAATGA	C-2<br>C-3	-2<br>-6	-24<br>-9
253	GTTGC-…-----------------GCCC…CGGGAAGCCAGAAG-------GAATGA	C-2<br>C-3	-2<br>-2	-24<br>-7
254	GTTGCT…TTGAGTTTCCCACAGAAGGC…GGT-…---------GTTCTAGGAATGA	C-2<br>C-3	+2<br>-16	+2<br>-14
255	GTTGCT…GG-----------------C…CGGGAAGCCAG--GGTTCTAGGAATGA	C-2<br>C-3	-5<br>-5	-23<br>-2
256	GTTGCT…TTGAG------AGAAGGCCC…CGGGAAGCCA-----TTCTAGGAATGA	C-2<br>C-3	+4<br>-6	-6<br>-5
257	GTTGCT…TTGAGTTTCCCAGAAGGCCC…CGGGAAGCCAGAATG-TCTAGGAATGA	C-2<br>C-3	 -3	 +1 /-1
258	GTTGC-…-----------------GCCC…CG------------------GGAATGA	C-2<br>C-3	-2<br>-14	-24<br>-18
259	GTTGCT…TTGAG----------GTGTC…CGGGAAGCCAGAA---------AATGA	C-2<br>C-3	-7<br>-3	-16<br>-9
260	GTTGC-…------------------CC…CGGGAAGCCA-----------GAATGA	C-2<br>C-3	-4<br>-6	-26<br>-11
261	GTTGCT…GGC-…----------GGCCC…GG--------…----------GAATGA	C-2<br>C-3	-1<br>-22	-18<br>-27
262	GTTGCT…TTGA------CAGAAGGCCC…CGG------------TTCTAGGAATGA	C-2<br>C-3	+5 -13 	-6<br>-12
263	GTTGC-…-----------------GCCC…CGGGAAGCCA------TCTAGGAATGA	C-2<br>C-3	-2<br>-6	-24<br>-6
264	GTTGCT…TTGAGTTTCCCAGAAGGCCC…CGGGAAGCCAGAA--------GAATGA	C-2<br>C-3	 -3	 -8
265	GGCC…GCTTG----------AAGGCCC…AGGC------…-----------…-CAC	C-2<br>C-3	-8<br>-21	-10<br>-338, +800
266	GTTGCT…TTGAG------------CCC…CGGGAAG------------AGGAATGA	C-2<br>C-3	-3<br>-9	-12<br>-12
267	TGTC-…----------------------------…-GAAG…TCGG-…----…-AG	C-2<br>C-3	-464<br>-13	-2,300<br>-18
268	GTTGCT…TTGA----------AGGCCC…GGTCGGGAAGC-…--TTCTAGGAATGA	C-2<br>C-3	+1<br>-8	-10<br>-7
269	GAAGCT…TTGAGTTTCCCAGAAGGCCC…GGTCGGGAAGCCAG-…-----GAATGA	C-2<br>C-3	 -5	 -10
526	GTTGC-…-----------------GCCC…GCCAGGTACA…CCTATTCTAGGAATGA	C-2<br>C-3	-2<br>-198	-24<br>+196
527	GTTGCT…GGC-…-------------CC…GGTCGGG…CAGAAGGTTCTAGGA-TGA	C-2<br>C-3	-4<br>+8	-21<br>-1
528	GTTGC-…-----------------GCCC…GGTCGGG…CAGAAGG------GAATGA	C-2<br>C-3	-2<br>-1	-24<br>-6

b) Genomic site D-1/3/4 (CTCF binding sites in *Wap* locus)

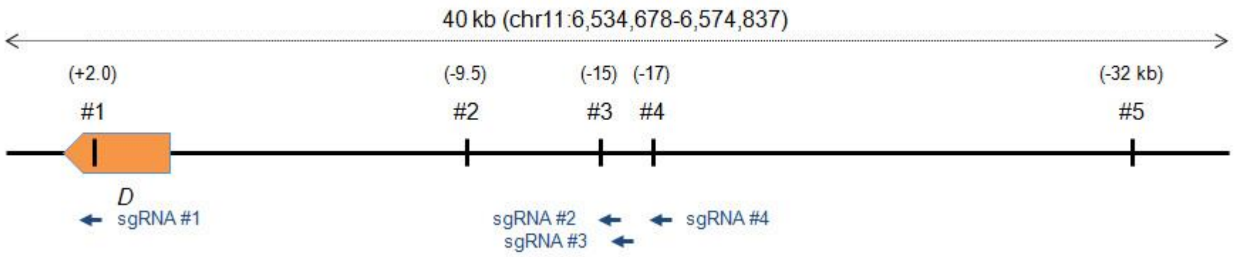

1) sgRNA sequence

Target site		sgRNA sequence	Co-injection (Yes/No)
D-1	sgRNA #1	5'-GGCACTGTAAAGAATTGGGC -3'	Yes
D-3	sgRNA #2	5'-GAAAGTTGTGAGCCACCAAG-3'	
	sgRNA #3	5'-GTACCTGTGCCCACCAGAAG-3'	
D-4	sgRNA #4	5'-AACTCAGTCTGTGGTAGATG-3'	

2) Target site

a. Site D-1

- Blue: Target sequence (CTCF binding region)
- Yellow: Target NGG sequence complemented to sgRNA
- Underline: sgRNA #1 binding site

CGAACTCAGAAATCTGCCTGCCTCTGCCTCCCAAGTACTGGGATCAAGGTGTGCGCCACCACTGCCCGGCGATTTAT
TTTTATACCTAAGTACACTGTAGCTGTCTTCAGACGCACCAGAAGAGGTCGTCAGATCTCATTACATATGGTTGTGA
GCCACCATGTGGTTGCTGGAATTTGAACTCAGGACCTTTGGAAGAGCAGTCCGTGCTCTTACCTGCTAAGCCATCTC
TCCAGCCCAATTCTTTACAGTGCCCCCATCACCCATAGACAGGTCGGTGAGACACGCCCGCCTGCAGGGGGCGCCCA
GGTGGACATGGGCAGCCGGATGTGAGCTCATGTTCTTAACTTTGAGCACTTTACTGTTTGCTCAGGTTCAGTGCCTA
AGGCACCATGTGGCCCATAGACTCACCAGGACTAGTTTTGAGTGCTAGAATGGTCTGTTCTCTCAGGTCCATTGTTC
ATTGAC

b. Site D-3

- Blue: Target sequence (CTCF binding region)
- Yellow: Target NGG sequence complemented to sgRNA
- **Bold**: sgRNA #2 binding site
- Underline: sgRNA #3 binding site

GCCCAGGACACAGGAAAAAAGGATAAAGAGCCTTTGAGCTAGGATACCCTCCTCCTCCTCCTCCTCCTCCTCC
TCTTCCTCCTCCTCCTCTTCTTCCTCCTCCTCCTCTCCTCCTTTTTCTTCCTGGAACTCACTCTGTAGACCAGGCTG
GCCTCAAACCCAGAAACCCACCTGCCTCTGCCTCCCAAGTGCTGGGATTAAAGGTGTGTGCCACCACTGCCCGGCCC
AGGTTTGTTTTTCAGGACCCT**CTTGGTGGCTCACAACTTTC**TGGAACTCATAGTTGCAAGGGGATCTGACATCCTCT
TCTGGTGGGCACAGGTACTGCATGCATGTGGTACACTGTCATACATGTAGACAAATCAACCAGATACATAAGTTAGA
AATGAAGAAACTGGGCTGGTGAGATGGCTCAGTGGGTAAGAGTACCCGACTGCTCTTCCGAAGGTCTGAAGTTCAAA
TCCCAGCAACCACATGGTGGCTCACAACCACCTGTAATGAGATCTGATGTCCTCTTCTGGTG

c. Site D-4

- Blue: Target sequence (CTCF binding region)
- Yellow: Target NGG sequence complemented to sgRNA
- Underline: sgRNA #4 binding site

CCCTGACTTGGTCATTATGCATTGTATTGAAGAGCCTCATTGGACCTTATAAATATGTATTATTACTATGTGTTAAT
TAAAATACATTTTAAAAAGAAGACAGAATTGGATAGTGAGTTGTAGGCTGGGTCAGAAACATTCCTTTATGTAATTC
TCCCTTCTGCAAGTATGTTAAGTCTACCTCATCTACCACAGACTGAGTTACTTAAATGTTATTCCAGCAGGGGGCGC
CAAATCATCAGGAATGCCTCTGAGGCAGTCTCGTTGGATGGGAGAAGAAACTAATCTCTGATGCTTCTTGAGTGTGT
GTGTGTGTGTGTGTGTGTGTGTGTGTATGTGTGATAGGTCTGGTTAGTATATCTGTGTGTGATTTGTGTATGTGTCT
CATATGTGTGTGGTTGTGTGTATCATGTGTAAATGTGTATGTGTATTGTATATGTGTGTACATATGTGCGTGCTGTA
TGTGCAACGTGCATATGTATGTGTGCACACGTGCTCGTG

3) Mutated sequence

- Yellow: Target NGG sequence complemented to sgRNA
- --- : deletion sequence, red: insertion or point mutation
- +1: putative Cas9 cutting site (3 nucleotide upstream of the nearest target NGG sequence)
- - bp: deletion size, + bp: insertion size, -: unknown (only sequenced from one end)

No.	Sequence	Site	Mutation start point	Mutation size (bp)
WT	D-1 (#1) D-3 (#2) D-3 (#3) D-4 (#4)<br>TAC…AGCCC…CAG…GGACCCTCT…CAC…ATCCTCTTCTG…GTCTACCTCAT…GAC			
8281	TAG…AGCCC…CAG…GGACCCTC-…-AC…ATCCT-----G…G-------T…GAC	D-3<br>D-4	-8, -2<br>+1	-10, -5<br>-9
8283	AG-…-----…-AG…GGACCCTCT…CAC…ATCC----CTG…GTCTACCT-…-C…AC	D-1<br>D-3<br>D-4	-8<br>-3<br>-6	-43<br>-4<br>-9

c) Genomic site D-1/2/3/4/5 (CTCF binding sites in *Wap* locus)

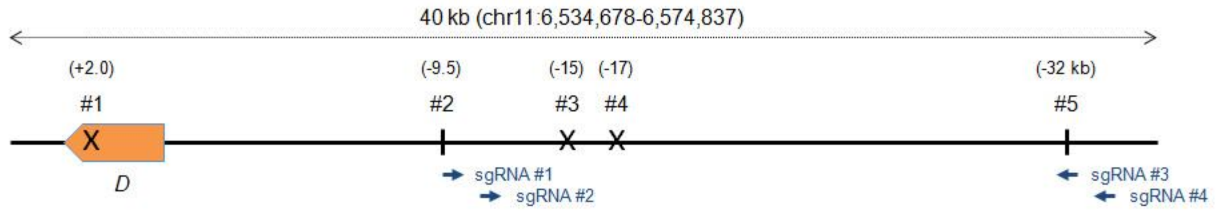

*** sgRNAs targeting site D-2 and D-5 were injected into zygotes from mice carrying deletion of D-1/3/4.*

1) sgRNA sequence

Target site		sgRNA sequence	Co-injection (Yes/No)
D-2	sgRNA #1	5'-TGAGTTTCCCATATGAACTC-3'	Yes
	sgRNA #2	5'-TCCTGATGGCCCTCTCCTGC-3'	
D-5	sgRNA #3	5'-GCTCAGTGGAGAACCAATCT-3'	
	sgRNA #4	5'-TGGGTGTATGTAAGTAAGTG-3'	

2)Target site

a. Site D-2

- Blue: Target sequence (CTCF binding region)
- Yellow: Target NGG sequence complemented to sgRNA
- **Bold**: sgRNA #1 binding site
- Underline: sgRNA #2 binding site

GGATAGCTCTTGCCAGGTGTTTCTTCTGCTGAGAATGATGAGCGGCCCTGACACCTCAAAGCCGCTTTGCTCCTTTGCACATGGATCTCAGAAAAAGCCTC**TGAGTTTCCCATATGAACTC**AGGAGACCCAACCCAACCCAACCCAACCCAACCCAACCCAACCCAACCCAACCCAACCCAACAAACACCCAAACCGAGAGAAATAACAAAACGGCTCACAGCTCCCTCCACTGGCCCATATGAGTTACTGCAGCTGCTTTATCAAAAAACAGCTTCTGGGTCCACAGCAGAGCCAGGCTTTCTTCCAACCTTTCTAGGTCAAAGCCAAGTCCTGAGCTCCCAGTTCTTTTCCTTGCTTTCTCCACGATACATGTCATCCCTGTTGACCCAGCTTGTTCAGCCACACCGGTTTCCTGATGGCCCTCTCCTGCAGGAACCTCTCGCTGAGGTCAAGTGACTACA

b. Site D-5

- Blue: Target sequence (CTCT binding region)
- Yellow: Target NGG sequence complemented to sgRNA
- **Bold**: sgRNA #3 binding site
- Underline: sgRNA #4 binding site

TTCTTAGCTTTCTTCTCTCAGTGTACAGCCTCCTCCCGACCTGCCA**GCTCAGTGGAGAACCAATCT**TATTGGCTGATCATGCTTTCACATAGCATTTGCAGAAGTGGGGCTGGGTGGGGAAGGAGCCTCTGTCCTTCTGGATTCATTGTCCAAATGGACAGAGCTCCTACCGTATGTGGCTCAGAGAATGAGATGTATCTGTTCCTCACAGAGGAAAGCTGTAGGCACAGACTGGGAGCTGGTGTTGAAGGGTGGATACTTCCCCCTTGAGGTTGAATGGGGTAGAGTTTCTGTAACTGGAGCTGGGGCAGCGGGGATCAGTTTGTGACTCAGTGTTACAGACTCACTGCTCCAAGATATTCTCTCTCTCTCTCTCTCTCTCTCTCTTGCTCTCTCTCTCTCTCTCTGTTTGCACACATGTGTGCACATGCATGCATGTGTTTGTATGTGTCCTTGGGTGTATGTAAGTAAGTGTGTGGGCATGTGTGCACACATATGCACATGTGCATGTATATACAGAGAC

3) Mutated sequence

- Blue: Target sequence (CTCF binding region)
- Yellow: Target NGG sequence complemented to sgRNA
- --- : deletion sequence, red: insertion or point mutation
- +1: putative Cas9 cutting site (3 nucleotide upstream of the nearest target NGG sequence)
- - bp: deletion size, + bp: insertion size, -: unknown (only sequenced from one end)

No.	Sequence	Mutation start point	Mutation size (bp)
WT	D-2(#1) 293 bp D-2(#2) D-5(#3) 387 bp D-5(#4)<br>AACTCAGGAGACC…CCTGCAGGAACCT…ACCTGCCAGCTCA…TGTGTCCTTGGGT		
2215A	AAG-…--------…-------------…-------------…--------…-TAC	-21	23,425
2215B	AACTCAGGAGACC…TCT-…--------…-------------…--------…-TGT	-2	22,247
2216A	TAT-…--------…-------------…-------------…--------…-TGT	-3	22,554
2216B	AAG-…--------…-------------…--------…-CCA…TGTGTCCTTGGGT	-21	22,171
2217A	TAA-…--------…-------------…-------------…--------…-TAT	-362	22,915
2217B	TAA-…--------…-------------…--------…-AAT…TGTGTCCTTGGGT	-624	22,776
2217C	AGT-…--------…-------------…--------…-CTC…TGTGTCCTTGGGT	-12	22,150
2218A	GAG-…--------…-------------…-------------…--------…-AAT	-13	23,503
2218B	TAA-…--------…-------------…-------------…--------…-GTA	-334	22,895
2218C	TTC-…--------…-------------…--------…-CAG…TGTGTCCTTGGGT	-92	22,232

2219	AAA-…--------…-------------…--------…-AAC…TGTGTCC-…-TTG	-1,191;<br>+5 (D-5 #4)	23,339;<br>344
2220A	CTT-…--------…-------------…-------------…--------…-GTG	-293	22,843
2220B	ATG-…--------…-------------…-------------…--------…-TAA	-2	22,559
2220C	CAT-…--------…-------------…--------…-AAC…TGTGTCCTTGGGT	-5	22,153
2221A	ATG-…--------…-------------…-------------…--------…-GTA	-2	22,558
2222A	ATG-…--------…-------------…-------------…--------…-CTT	-257	23,987
2222B	CCT-…---…-TCT…TGA…---------…-------------…--------…-GGA	-18(D-2 #1);<br>-11	240;<br>22,843
2222C	ACC-…--------…-------------…--------…-ACC…TGTGTCCTTGGGT	-135	22,284
2223A	CAC-…--------…-------------…-------------…--------…-AAG	-587	23,520
2223B	AAC-…--------…-------------…-------------…--------…-GAA	-612	23,287
2223C	G-…-T…GGAGACC…TGG-…--------…-------------…--------…-TGG	+2 (D-2 #1);<br>-8	118;<br>22,483
2223D	A------------…-------------…--------…-GAA…TGTGTCCTTGGGT	-1	22,147

Supplementary Reference

1. Sung YH*, et al.* Highly efficient gene knockout in mice and zebrafish with RNA-guided endonucleases. *Genome Res* **24**, 125-131 (2014).
